# Hidden inequities in universal health coverage: determinants of health insurance underutilization in Indonesia

**DOI:** 10.1186/s12889-026-26841-3

**Published:** 2026-03-11

**Authors:** Sujarwoto Sujarwoto, Holipah Holipah, Asri Maharani

**Affiliations:** 1 Department of Public Administration, Universitas Brawijaya, Malang, Indonesia; 2 Department of Public Health, Faculty of Medicine, Universitas Brawijaya, Malang, Indonesia; 3 Division of Nursing, Midwifery and Social Work, School of Health Sciences, University of Manchester, Jean McFarlane Building Oxford Road, Manchester, M13 9PL UK

**Keywords:** JKN, Health insurance, Underutilisation, Equity, Indonesia

## Abstract

**Background:**

Universal health coverage (UHC) requires not only insurance enrolment but also effective use of coverage. In Indonesia, despite near-universal enrolment in the National Health Insurance programme (*Jaminan Kesehatan Nasional*, JKN), many insured individuals do not use their insurance when seeking care. Evidence on the extent and determinants of this underutilisation remains limited.

**Methods:**

We analysed nationally representative data from the 2023 National Socioeconomic Survey (SUSENAS), including insured individuals who reported outpatient care in the past month or inpatient care in the past 12 months. Insurance underutilisation was defined as the non-use of JKN during a healthcare encounter among those insured. Guided by Andersen’s Behavioral Model, we estimated random-effects logistic regression models for outpatient and inpatient care, incorporating individual, household, and district-level health system factors and accounting for clustering at the district level.

**Results:**

Among insured individuals, 48.3% did not use JKN for outpatient care, compared with 11.0% for inpatient care. In adjusted models, outpatient non-use was higher among self-employed (aOR 1.43), casual (aOR 1.54), and unpaid family workers (aOR 1.42) than among those not working, and increased with income (highest vs. lowest quartile: aOR 1.34). Rural residents were more likely to underutilise outpatient insurance (aOR 1.47), while higher education was protective (university vs. elementary: aOR 0.77). Greater hospital (aOR 0.28) and primary care density (aOR 0.86) reduced outpatient non-use. For inpatient care, non-use remained associated with self-employment (aOR 1.29), high income (aOR 1.56), rural residence (aOR 1.25), and lower hospital density (aOR 0.21). Between-district differences explained 21% of outpatient and 14% of inpatient variation.

**Conclusions:**

Substantial inequities persist in the effective use of JKN, particularly for outpatient care. Addressing labour-market constraints, opportunity costs, administrative barriers, and local service capacity is essential to translate coverage into equitable access.

**Supplementary Information:**

The online version contains supplementary material available at 10.1186/s12889-026-26841-3.

## Introduction

Achieving equitable access to healthcare remains a central challenge for many low- and middle-income countries (LMICs), even after substantial expansion of universal health coverage (UHC). National health insurance schemes are intended to remove financial barriers to care, yet insurance coverage does not automatically translate into service use. A large body of literature documents persistent socioeconomic and geographic inequalities in healthcare utilisation among the general population [1–3]. Far less attention has been paid to inequities within insured populations, where individuals who are formally covered do not use their insurance when accessing healthcare services. This phenomenon—health insurance underutilisation among the insured—represents a distinct and underexamined dimension of inequity, shifting the analytical focus from who is covered to who effectively benefits from coverage [4–6].

Indonesia offers an important case for examining this coverage–use gap. Since the launch of the National Health Insurance programme (Jaminan Kesehatan Nasional, JKN) in 2014, Indonesia has established one of the world’s largest single-payer systems, covering more than 240 million people [7]. Through premium subsidies for poorer households and mandatory enrolment of formal-sector workers, JKN has substantially expanded financial protection [8]. Most existing studies assess JKN’s impact on healthcare utilisation or financial risk protection in the population as a whole [9–12]. While these studies demonstrate improvements in access and reductions in catastrophic spending, they do not directly address whether insured individuals consistently use JKN when seeking care.

Emerging evidence suggests that many insured Indonesians bypass JKN during healthcare encounters, particularly for outpatient services. Studies focusing on insured populations indicate that rural residents, informal workers, and socioeconomically disadvantaged groups are more likely to pay out-of-pocket or seek non-contracted providers despite being insured [7, 9, 13, 14]. Importantly, this behaviour reflects non-use of insurance rather than non-use of healthcare. Underutilisation may arise from administrative complexity, weak referral systems, limited provider availability, perceived low quality of contracted services, or patient preferences. Framing this issue as a disconnect between coverage and effective use clarifies that insurance alone is insufficient to ensure equitable access.

Research on insurance underutilisation remains limited. Few studies conceptualise non-use of insurance as a distinct outcome of inequity, and none have examined its magnitude and determinants using recent nationally representative data across both outpatient and inpatient care. The interaction between individual characteristics and contextual health system factors, such as facility density and local infrastructure, also remains poorly understood.

This study addresses these gaps using data from the 2023 National Socioeconomic Survey (SUSENAS). Guided by Andersen’s Behavioural Model of Health Services Use [13, 15], we examine how predisposing, enabling, and need factors, together with district-level health system characteristics, shape patterns of JKN underutilisation among insured individuals. We extend Andersen’s framework by explicitly incorporating *prohibiting factors*, defined as structural and administrative barriers that impede insurance use despite formal coverage. The study aims to quantify the prevalence of JKN non-use and to identify key demographic, socioeconomic, and health system determinants of inequitable insurance utilisation.

By conceptualising insurance underutilisation as a form of hidden inequity within UHC, this study contributes to the evidence base on effective access to care. It provides policy-relevant insights to strengthen JKN implementation and offers lessons for other LMICs seeking to move beyond nominal coverage towards the equitable realisation of UHC.

## Methods

### Study design and data sources

This study employed a cross-sectional design using secondary data from a nationally representative survey administered by the Indonesian Central Bureau of Statistics (BPS): the 2023 National Socioeconomic Survey (SUSENAS) wave. The SUSENAS provides detailed household- and individual-level information on demographic, economic, education, and health indicators, covering approximately 1.25 million individuals across 514 districts and 38 provinces [16, 17].

### Study population and sampling

The National Socioeconomic Survey (SUSENAS) employs a multi-stage stratified random sampling design to ensure national representativeness. Primary sampling units, or enumeration areas (EAs), are selected using probabilities proportional to size, followed by systematic selection of households within each EA. Stratification is based on province, urban–rural classification, and household income quintile, minimising sampling bias and enhancing precision [16].

The analytic sample was derived through a stepwise restriction of the original SUSENAS dataset (Figure S1, Supplementary files). The full survey comprised 1,223,377 individuals, representing a weighted national population of 276,786,794. From this base, the analysis was restricted to respondents who reported owning any form of public health insurance, i.e., JKN PBI, JKN non-PBI, or Jamkesda, yielding 886,695 insured respondents, corresponding to 193,413,674 individuals at the national level. Within this insured population, two care-specific analytic samples were constructed based on recent healthcare utilisation. For outpatient care, the final sample included 84,072 respondents, representing 19,108,067 individuals nationally. For inpatient care, the analytic sample comprised 32,650 respondents, corresponding to 7,635,331 individuals after weighting. These sequential restrictions ensured that the study focused specifically on insured individuals with observed healthcare use, allowing valid estimation of insurance non-use among those eligible to activate JKN during outpatient and inpatient encounters.

### Dependent variable

The dependent variable was health insurance underutilisation, defined as non-use of JKN among insured individuals when accessing healthcare services. It was operationalised as a binary outcome, coded as 1 for respondents enrolled in JKN who did not use the insurance for a reported healthcare encounter, and 0 for those who did.

Separate dependent variables were constructed for outpatient and inpatient care to reflect differences in recall period, conditioning, and utilisation pathways in the SUSENAS survey. Outpatient non-use was defined as respondents who reported a health problem and sought outpatient care in the past one month, but did not activate insurance for routine or ambulatory services. In contrast, inpatient non-use was defined among respondents who reported any hospitalisation in the past 12 months, irrespective of recent illness reporting, reflecting bypass of JKN for higher-acuity care.

These outcomes were analysed separately throughout the study, recognising that outpatient and inpatient services differ in clinical severity, decision-making processes, and administrative requirements. To minimise potential misclassification, additional analyses excluded respondents reporting inactive JKN cards, ensuring that non-use primarily reflected behavioural, structural, or health system barriers rather than administrative status. This operationalisation is consistent with prior studies on insurance utilisation and aligns with Andersen’s behavioural model of healthcare use [13].

### Independent variables

Independent variables were selected a priori based on Andersen’s behavioural model of health service use and prior evidence on health insurance utilisation in Indonesia, and were grouped into individual, household, contextual, and health system supply domains.

Individual characteristics included sex (male/female), age group, educational attainment, and employment status. Age was categorised into seven groups reflecting life-course stages: infants (< 1 year), children (1–9 years), early adolescents (10–14 years), late adolescents (15–19 years), young adults (20–24 years), adults (25–59 years), and older persons (≥ 60 years) [18]. Educational attainment was classified as primary (elementary or less), junior secondary, senior secondary, diploma, and university. Employment status captured labour-market position and was categorised as not working, self-employed, employee, casual worker, or unpaid family worker.

Household characteristics comprised household size, measured as a continuous variable indicating the number of household members, and household economic status, proxied by per capita household income quartiles (Q1–Q4), with Q1 representing the lowest income group. These quartiles were constructed within the analytical sample of insured individuals to capture relative socioeconomic position among JKN beneficiaries.

Contextual factors included place of residence (urban vs. rural) and health need. Health need was measured using a self-reported indicator of whether the respondent experienced a health problem that affected daily activities during the reference period, capturing perceived morbidity and functional limitation.

Health system supply variables were measured at the district level to capture local service availability and capacity. These included hospital density, primary care facility density, doctor density, and overall health worker density, each operationalised as ratios per district population. These indicators were linked to individual respondents based on district of residence and entered as continuous variables in the regression models.

All independent variables were included in the fully adjusted multivariable models for outpatient and inpatient care. Reference categories were defined as infants for age, elementary or less for education, not working for employment status, and the lowest income quartile (Q1) for household income.

### Statistical analysis

We first conducted weighted descriptive analyses to characterise patterns of non-use of JKN insurance among insured respondents for outpatient and inpatient care, controlling for sociodemographic, socioeconomic, household, and district-level health system characteristics. Differences in distributions between outpatient and inpatient samples were examined to assess whether non-use was more concentrated in specific population groups or care settings. Geographic variation in non-use was visualised using district-level choropleth maps, enabling identification of spatial clustering and regional inequalities. To explore mechanisms underlying non-use, we further examined reported reasons for not using JKN through heatmaps stratified by key sociodemographic characteristics, allowing comparison of access-related versus preference-driven explanations across outpatient and inpatient services.

To identify factors independently associated with non-use of JKN, we estimated random-effects logistic regression models separately for outpatient and inpatient care. Model A estimated unadjusted associations using single-domain specifications, while Model B included all individual, household, and district-level covariates simultaneously. Random intercepts at the district level accounted for clustering and unobserved contextual heterogeneity. Results are reported as odds ratios (ORs) and adjusted odds ratios (aORs) with 95% confidence intervals. Intraclass correlation coefficients (ICCs) were calculated to quantify the proportion of variance attributable to between-district differences. As a sensitivity analysis, respondents reporting inactive JKN cards were excluded to reduce misclassification of insurance status; patterns from these restricted models were compared with the main results to assess robustness and to distinguish behavioural and structural barriers from administrative ineligibility. Statistical significance was defined as *p* < 0.05, and analyses were performed in Stata version 19.0.

## Results

### Socio-demographic, socioeconomic, household, and health system characteristics

Table 1 summarises key descriptive patterns in JKN non-use among insured respondents. Non-use is more common in outpatient than inpatient care, suggesting that insurance is less consistently activated for routine services and more likely to be used when hospitalisation is required. Men account for a larger share of non-users than women, and non-use is concentrated among adults of working age, with smaller contributions from children, adolescents, and older persons. Lower educational attainment, particularly elementary and junior secondary schooling, characterises the largest share of non-users, although inpatient non-use includes a relatively higher proportion of individuals with senior secondary and university education. Non-use is most frequent among those not working and the self-employed, while casual and unpaid family workers contribute smaller shares. Across income quartiles, non-use is broadly distributed without marked concentration at either end of the income spectrum, and is more prevalent among urban than rural residents in absolute terms. Most non-users report health complaints affecting daily activities, and non-use is observed across districts with varying levels of health system supply, indicating that bypassing insurance is not limited to areas with constrained service availability.

**Table 1:** Demographic, socioeconomic, household, and health system characteristics of individuals with non-use of JKN insurance

Variables	Outpatient samples (*N* = 19,108,067)			Inpatient samples (*N* = 7,635,331)		
	*N*	% or Mean (SD)	Min-Max	*N*	% or Mean (SD)	Min-Max
Non-use of JKN insurance	7,101,106	48.32%		7,635,331	11.03%	
Sex						
Male	10,653,800	49.67%		4,678,396	61.27%	
Female	8,454,267	49.67%		2,956,935	38.73%	
Age group						
Infants (< 1 year) (ref)	2,016,526	30.72%		772,547	10.12%	
Children (1–9 year)	1,651,491	28.10%		339,936	4.45%	
Early adolescent (10–14 year)	1,151,156	23.79%		234,532	3.07%	
Late adolescent (15–19 year)	859,704	20.73%		319,629	4.19%	
Young adult (20–24 year)	711,607	18.94%		520,478	6.82%	
Adult (25–59 year)	7,997,616	49.33%		3,880,433	50.82%	
Older person (60 + year)	4,719,966	43.13%		1,567,776	20.53%	
Educational attainment						
Elementary or less	10,919,826	49.49%		3,448,446	45.16%	
Junior secondary	2,766,852	35.19%		1,177,466	15.42%	
Senior secondary	3,835,390	40.05%		1,952,086	25.57%	
Diploma	282,834	12.08%		195,698	2.56%	
University	1,303,164	25.21%		861,635	11.28%	
Employment status						
Not working	11,156,843	49.29%		4,692,832	61.46%	
Self-employed	3,750,159	39.72%		1,252,684	16.41%	
Employee	2,665,818	34.65%		1,270,394	16.64%	
Casual worker	698,600	18.77%		170,650	2.24%	
Unpaid family worker	836,647	20.46%		248,771	3.26%	
Household characteristics						
Household size (members)	48,194,729	2.52 (SD = 1.71)	1–9	19,222,373	2.52 (SD = 1.65)	1–9
Household income quartile						
Q1 (lowest)	4,878,484	25.53%		1,934,907	25.34%	
Q2	4,697,216	24.58%		1,856,741	24.32%	
Q3	4,679,143	24.49%		1,876,672	24.58%	
Q4 (highest)	4,853,223	25.40%		1,967,010	25.76%	
Place of residence						
Urban	12,041,638	63.01%		4,949,153	64.82%	
Rural	7,066,429	36.99%		2,686,178	35.18%	
Health complaint affecting daily activities						
No	7,018,521	36.73%		3,682,108	48.22%	
Yes	12,089,546	63.27%		3,953,223	51.78%	
Health system supply (district level)						
Hospital ratio	2,394,642	0.10 (SD = 0.10)	0-0.8	1,006,183	0.13 (SD = 0.10)	0-0.8
Primary care facility ratio	23,752,493	1.23 (SD = 1.23)	0.19–15.29	9,590,342	1.26 (SD = 0.19)	0.19–15.29
Doctor ratio	43,578,602	1.94 (SD = 1.94)	0.21–28.28	18,578,257	2.43 (SD = 2.19)	0.21–28.28
Health worker ratio	330,441,723	13.94 (SD = 13.94)	0.57–91.84	134,719,917	17.64 (SD = 13.80)	0.57–91.84

All estimates are weighted. JKN refers to Indonesia’s National Health Insurance. Non-use indicates respondents covered by JKN who did not use the insurance for the reported outpatient or inpatient service. Health system supply indicators are measured at the district level

### Geographic distribution of non-use of JKN

**Fig. 1:**
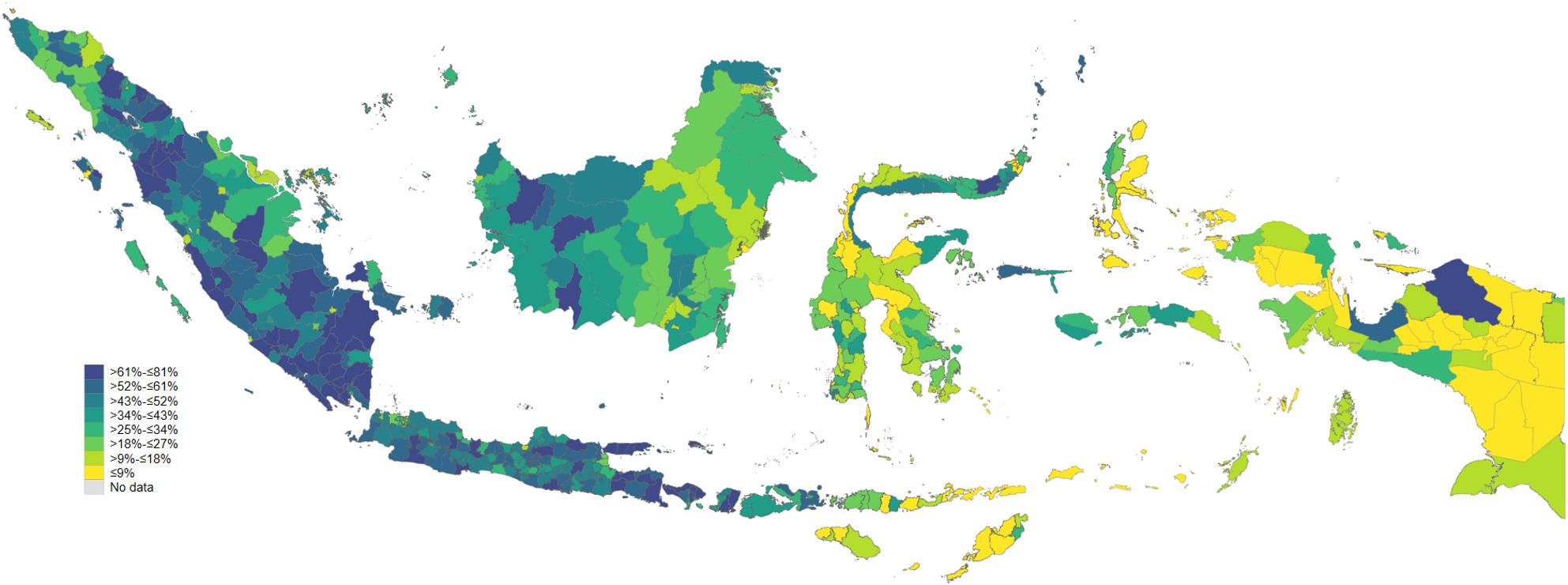
District-level prevalence of non-use of JKN insurance for outpatient services among insured individuals in Indonesia

Figure 1 shows pronounced geographic clustering of non-use of JKN for outpatient care across Indonesian districts, revealing clear regional inequalities. Higher levels of non-use are concentrated in much of Sumatra, Java, and parts of Kalimantan, where many districts fall into the upper categories, indicating that a substantial share of insured residents bypass JKN even in areas with relatively dense health service networks. In contrast, eastern Indonesia—particularly Maluku, Nusa Tenggara, and many districts in Papua—tends to exhibit lower levels of non-use, suggesting either more consistent reliance on JKN when seeking outpatient care or more limited alternatives to insured services. The spatial pattern does not align neatly with simple measures of remoteness: several urban and peri-urban districts display high non-use, while some more remote districts show lower levels. Overall, the map suggests that outpatient non-use of JKN is shaped not only by supply constraints, but also by local health system practices, administrative barriers, and patient preferences, resulting in marked subnational variation rather than a uniform national pattern.

### Reasons for non-use of JKN insurance for outpatient and inpatient care

**Fig. 2:**
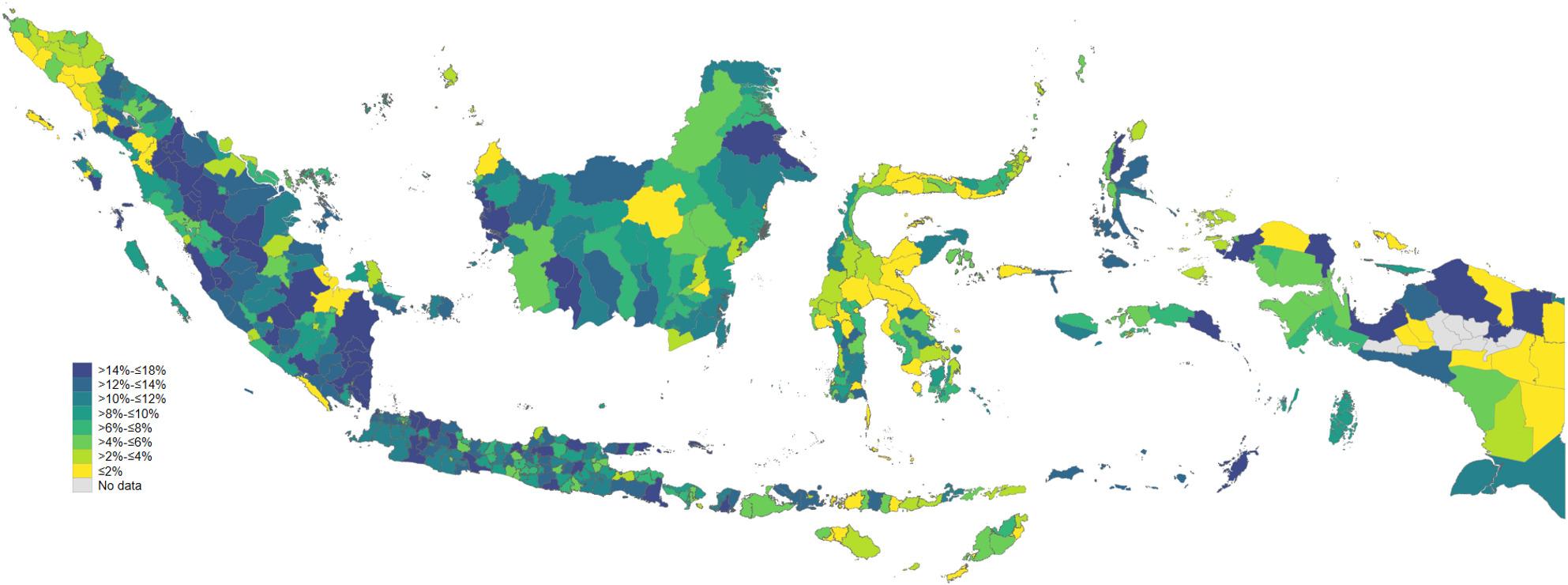
District-level prevalence of non-use of JKN insurance for inpatient services among insured individuals in Indonesia

Figure 2 shows a more compressed and lower overall level of non-use of JKN for inpatient care, with substantially less geographic dispersion than observed for outpatient services. Most districts fall within the lower categories, indicating that insured individuals are more likely to rely on JKN when hospitalisation is required. Nevertheless, distinct pockets of higher non-use persist, particularly across parts of Sumatra, Java, and Kalimantan, suggesting that bypassing JKN for inpatient care is not rare even in regions with relatively established hospital infrastructure. In contrast, many districts in eastern Indonesia, including Nusa Tenggara, Maluku, and large parts of Papua, exhibit very low non-use, consistent with stronger dependence on JKN for costly inpatient treatment and fewer viable alternatives outside the insurance scheme. Overall, the spatial pattern implies that while inpatient care creates a stronger pull toward JKN across the country, subnational differences remain and are likely driven by a combination of hospital availability, referral practices, perceived quality, and administrative or financial barriers, rather than geography alone.

**Fig. 3:**
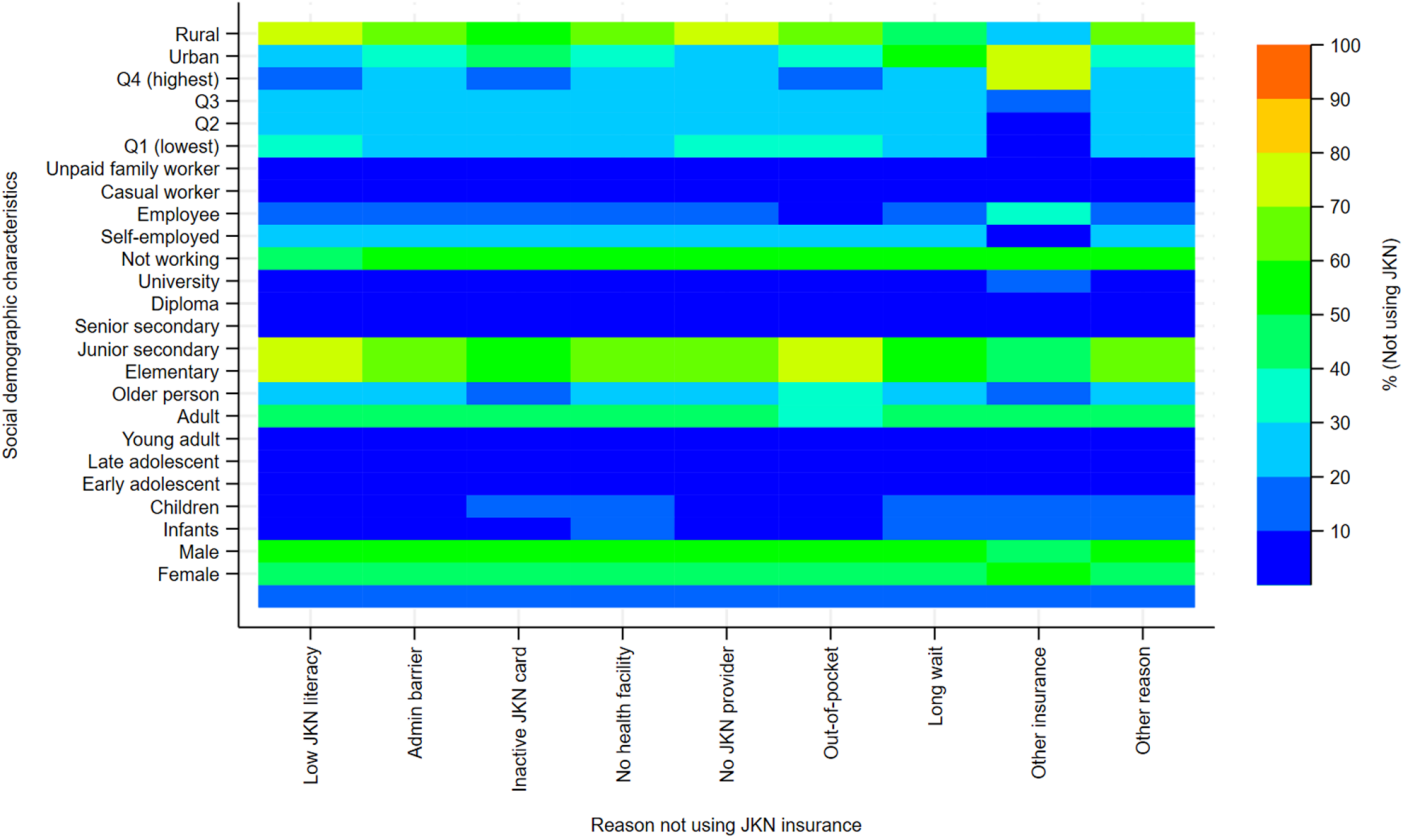
Reasons for non-use of JKN insurance for outpatient care by sociodemographic characteristics

The outpatient heatmap (Fig. 3) shows clear heterogeneity in reported reasons for not using JKN insurance across sociodemographic and socioeconomic groups. Structural and system-related barriers dominate among disadvantaged groups. Individuals in the lowest income quartile, those with elementary or less education, informal workers, and rural residents show high intensity for reasons related to administrative barriers, inactive JKN cards, lack of nearby facilities, and long waiting times. In contrast, higher-income and more educated groups display relatively stronger intensity for preference-based reasons, including perceived lack of need and use of non-JKN providers, suggesting discretionary non-use rather than access failure. Age gradients are pronounced: adults and older persons account for the highest intensity across most reasons, while children and adolescents contribute minimally, indicating that outpatient non-use is concentrated among populations with greater healthcare demand. Gender differences are small and consistent across reasons, indicating that outpatient non-use is not strongly gendered. Overall, the pattern highlights a dual dynamic in outpatient care: access and administrative constraints among socioeconomically disadvantaged groups, alongside preference-driven non-use among more advantaged populations, pointing to both supply-side and perceived-value challenges within JKN outpatient services.

**Fig. 4:**
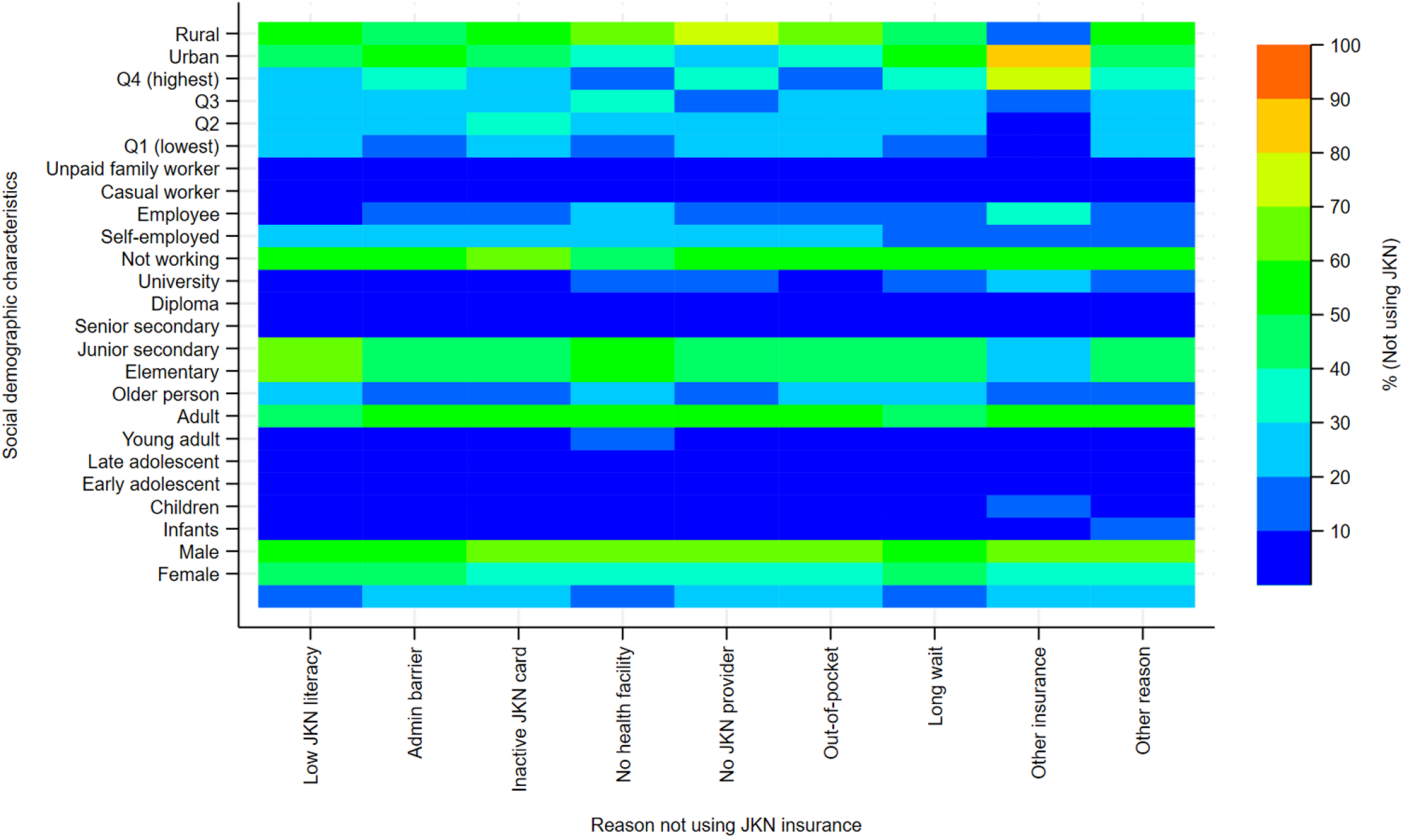
Reasons for non-use of JKN insurance for inpatient care by sociodemographic characteristics

The inpatient heatmap (Fig. 4) reveals a more concentrated and selective pattern of JKN non-use compared with outpatient care, with stronger clustering around specific socioeconomic groups and reasons. Non-use is predominantly observed among adults and older persons, indicating that even for higher-acuity services, JKN is not consistently utilised by groups with substantial healthcare needs. Socioeconomic gradients remain evident but are less uniform than in outpatient care. Higher-income and more educated groups show greater intensity for preference-related reasons—particularly use of alternative insurance and opting for non-JKN providers—suggesting deliberate bypass of JKN in inpatient settings. In contrast, lower-income groups and those with elementary or less education display higher intensity for system-related barriers, including administrative constraints, inactive cards, and facility-related limitations, although these are less widespread than in outpatient services. Rural–urban differences persist, with rural residents more frequently associated with access-related reasons, while urban residents show relatively higher intensity for insurance substitution. Gender differences are minimal across all reasons, indicating that inpatient non-use is not strongly gendered. Overall, the inpatient pattern suggests that non-use of JKN reflects a combination of selective opting-out among more advantaged groups and residual access and administrative barriers among disadvantaged populations, but with a narrower set of reasons than observed in outpatient care, consistent with the higher perceived necessity of inpatient services.

### Factors associated with non-use of JKN insurance for outpatient care

Table 2. Unadjusted and adjusted random-effects logistic regression of factors associated with non-use of JKN insurance for outpatient care. Model A presents unadjusted odds ratios (ORs) from single-domain random-effects logistic regression models. Model B presents fully adjusted ORs from a multivariable random-effects logistic regression. ORs are reported with 95% confidence intervals. Random intercepts were specified at the district level to account for clustering.

**Table 2:** Unadjusted and adjusted random-effects logistic regression of factors associated with non-use of JKN insurance for outpatient care (N individual = 84,072, N district = 514)

Predictor	Category / Unit	Model A		Model B	
		OR (95% CI)	*p*-value	aOR (95% CI)	*p*-value
Individual characteristics					
Sex	Female (vs. male)	1.05 (1.02–1.09)	0.001	1.00 (0.96–1.03)	0.799
Age group†	Infants (< 1 year) (ref)	1.00	—	1.00	—
	Children (1–9 year)	1.00 (0.93–1.07)	0.984	1.00 (0.93–1.07)	0.987
	Early adolescent (10–14 year)	0.86 (0.79–0.92)	< 0.001	0.89 (0.82–0.96)	0.003
	Late adolescent (15–19 year)	0.88 (0.81–0.96)	0.005	1.02 (0.93–1.13)	0.640
	Young adult (20–24 year)	0.87 (0.79–0.96)	0.008	0.88 (0.78–0.98)	0.024
	Adult (25–59 year)	0.72 (0.68–0.76)	< 0.001	0.65 (0.61–0.70)	< 0.001
	Older person (60 + year)	0.64 (0.60–0.68)	< 0.001	0.57 (0.54–0.61)	< 0.001
Education†	Elementary or less (ref)	1.00	—	1.00	—
	Junior secondary	0.88 (0.84–0.92)	< 0.001	0.89 (0.85–0.94)	< 0.001
	Senior secondary	0.78 (0.75–0.82)	< 0.001	0.78 (0.74–0.82)	< 0.001
	Diploma	0.62 (0.54–0.72)	< 0.001	0.65 (0.56–0.75)	< 0.001
	University	0.78 (0.73–0.83)	< 0.001	0.77 (0.71–0.83)	< 0.001
Employment†	Not working (ref)	1.00	—	1.00	—
	Self-employed	1.08 (1.04–1.12)	< 0.001	1.43 (1.36–1.50)	< 0.001
	Employee	1.01 (0.96–1.06)	0.630	1.39 (1.31–1.47)	< 0.001
	Casual worker	1.20 (1.10–1.31)	< 0.001	1.54 (1.40–1.69)	< 0.001
	Unpaid family worker	1.17 (1.09–1.26)	< 0.001	1.42 (1.31–1.53)	< 0.001
Household size	Per additional member	1.04 (1.03–1.05)	< 0.001	1.03 (1.02–1.05)	< 0.001
Income per capita quartile†	Q1 (lowest) (ref)	1.00	—	1.00	—
	Q2 (vs. Q1)	1.13 (1.08–1.19)	< 0.001	1.15 (1.10–1.21)	< 0.001
	Q3	1.09 (1.04–1.14)	0.001	1.14 (1.09–1.19)	< 0.001
	Q4 (highest)	1.18 (1.12–1.24)	< 0.001	1.34 (1.27–1.40)	< 0.001
Residence	Rural (vs. urban)	1.47 (1.42–1.53)	< 0.001	1.47 (1.41–1.53)	< 0.001
Health need	Health problem affecting daily activities (yes vs. no)	1.07 (1.04–1.11)	< 0.001	1.05 (1.02–1.09)	0.003
District characteristics					
Hospital density	Per unit increase	0.09 (0.04–0.19)	< 0.001	0.28 (0.12–0.66)	0.004
Primary care density	Per unit increase	0.85 (0.81–0.88)	< 0.001	0.86 (0.82–0.90)	< 0.001
Doctor density	Per unit increase	0.90 (0.86–0.94)	< 0.001	0.98 (0.94–1.03)	0.525
Health worker density	Per unit increase	0.98 (0.98–0.99)	< 0.001	0.99 (0.99–1.00)	0.015

Outcome: non-use of JKN insurance

Model A: unadjusted random-effects logistic regression (one predictor at a time). Model B: fully adjusted random-effects logistic regression including all covariates shown

Model B statistics: Wald χ²=984.83; intraclass correlation (*ρ*) = 0.205 (95% CI 0.182–0.229); likelihood-ratio test for random effects *p* < 0.001

*OR* odds ratio, *aOR* adjusted odds ratio, *CI* confidence interval

† Reference categories: age group = infants; education = elementary or less; employment = not working; income = lowest quartile (Q1). Models estimated using random-effects logistic regression with individuals clustered within 514 districts

Among insured respondents, non-use of JKN for outpatient care was strongly patterned by socioeconomic status, employment, and local health system context. After adjustment, sex was not associated with outpatient non-use (female vs. male: aOR 1.00, 95% CI 0.96–1.03). Clear age gradients were observed: adults aged 25–59 years and older persons were substantially less likely to not use JKN compared with infants (aOR 0.65, 95% CI 0.61–0.70; and aOR 0.57, 95% CI 0.54–0.61, respectively). Higher educational attainment was consistently protective, including university education (aOR 0.77, 95% CI 0.71–0.83). Employment status showed the strongest associations. Casual workers, self-employed individuals, and unpaid family workers had markedly higher odds of non-use than those not working (aORs ranging from 1.42 to 1.54). Non-use also increased with household size (aOR 1.03 per additional member, 95% CI 1.02–1.05) and across income quartiles, reaching the highest odds in the top quartile (aOR 1.34, 95% CI 1.27–1.40). Rural residents were significantly more likely to not use JKN (aOR 1.47, 95% CI 1.41–1.53). At the district level, higher hospital and primary care facility density were associated with lower non-use, whereas doctor density was not. The intraclass correlation coefficient indicated that approximately 21% of the variance in outpatient non-use was attributable to between-district differences.

### Factors associated with non-use of JKN insurance for inpatient care

Table 3. Unadjusted and adjusted random-effects logistic regression of factors associated with non-use of JKN insurance among the inpatient sample. Model A presents unadjusted odds ratios (ORs) from single-domain random-effects logistic regression models. Model B presents fully adjusted ORs from a multivariable random-effects logistic regression. ORs are reported with 95% confidence intervals. Random intercepts were specified at the district level to account for clustering.

**Table 3:** Unadjusted and adjusted random-effects logistic regression of factors associated with non-use of JKN insurance among the inpatient sample (N individuals = 32,650, N districts = 506)

Predictor	Category / Unit	Model A		Model B	
		OR (95% CI)	*p*-value	aOR (95% CI)	*p*-value
Individual characteristics					
Sex	Female (vs. male)	0.99 (0.91–1.08)	0.859	0.97 (0.89–1.06)	0.502
Age group†	Infants (< 1 year) (ref)	1.00	—	1.00	—
	Children (1–9 years)	1.02 (0.82–1.26)	0.884	1.02 (0.82–1.27)	0.875
	Early adolescent (10–14 years)	0.91 (0.71–1.17)	0.475	0.90 (0.70–1.17)	0.434
	Late adolescent (15–19 years)	1.24 (0.99–1.54)	0.057	1.22 (0.96–1.55)	0.111
	Young adult (20–24 years)	0.95 (0.77–1.17)	0.649	0.94 (0.74–1.18)	0.577
	Adult (25–59 years)	0.95 (0.82–1.09)	0.452	0.89 (0.75–1.07)	0.213
	Older person (60 + years)	0.88 (0.75–1.03)	0.111	0.81 (0.68–0.96)	0.018
Education†	Elementary or less (ref)	1.00	—	1.00	—
	Junior secondary	1.02 (0.91–1.15)	0.685	1.02 (0.90–1.15)	0.793
	Senior secondary	1.01 (0.92–1.12)	0.802	0.99 (0.88–1.11)	0.822
	Diploma	0.79 (0.59–1.05)	0.109	0.79 (0.59–1.07)	0.125
	University	0.91 (0.79–1.05)	0.202	0.92 (0.78–1.08)	0.290
Employment†	Not working (ref)	1.00	—	1.00	—
	Self-employed	1.22 (1.10–1.35)	< 0.001	1.29 (1.15–1.45)	< 0.001
	Employee	0.90 (0.80–1.01)	0.079	0.93 (0.81–1.07)	0.306
	Casual worker	1.17 (0.91–1.51)	0.220	1.25 (0.96–1.63)	0.097
	Unpaid family worker	1.30 (1.07–1.58)	0.009	1.31 (1.07–1.59)	0.009
Household size	Per additional member	1.01 (0.98–1.04)	0.452	1.02 (0.99–1.05)	0.227
Income per capita quartile†	Q1 (lowest) (ref)	1.00	—	1.00	—
	Q2	1.12 (0.99–1.26)	0.061	1.14 (1.02–1.29)	0.027
	Q3	1.08 (0.96–1.22)	0.193	1.13 (1.00–1.28)	0.047
	Q4 (highest)	1.43 (1.27–1.60)	< 0.001	1.56 (1.38–1.77)	< 0.001
Residence	Rural (vs. urban)	1.22 (1.11–1.34)	< 0.001	1.25 (1.13–1.39)	< 0.001
Health need	Health problem affecting daily activities	0.90 (0.83–0.97)	0.008	0.88 (0.81–0.96)	0.002
District characteristics					
Hospital density	Per unit increase	0.09 (0.04–0.19)	< 0.001	0.21 (0.08–0.50)	0.001
Primary care density	Per unit increase	0.91 (0.88–0.95)	< 0.001	1.00 (0.95–1.05)	0.876
Doctor density	Per unit increase	0.88 (0.85–0.92)	< 0.001	0.97 (0.93–1.02)	0.248
Health worker density	Per unit increase	0.98 (0.98–0.99)	< 0.001	0.98 (0.98–0.99)	< 0.001

Outcome: non-use of JKN insurance

Model A: unadjusted random-effects logistic regression (one predictor at a time)

Model B: fully adjusted random-effects logistic regression including all covariates shown. Model B statistics: Wald χ²= 228.73; intraclass correlation (*ρ*) = 0.140 (95% CI 0.116–0.167); likelihood-ratio test for random effects *p* < 0.001

*OR* odds ratio, *aOR* adjusted odds ratio, *CI* confidence interval

† Reference categories: age group = infants; education = elementary or less; employment = not working; income = lowest quartile (Q1). Models estimated using random-effects logistic regression with individuals clustered within 506 districts

Among insured respondents, non-use of JKN for inpatient care was patterned primarily by socioeconomic position, employment status, place of residence, and health system capacity. In the adjusted model, sex was not associated with inpatient non-use (female vs. male: aOR 0.97, 95% CI 0.89–1.06). Age differences were generally modest; however, older persons (≥ 60 years) were significantly less likely to not use JKN compared with infants (aOR 0.81, 95% CI 0.68–0.96), while other age groups did not differ meaningfully after adjustment. Educational attainment was not independently associated with inpatient non-use once other factors were controlled. Employment status remained a key determinant: self-employed individuals and unpaid family workers had substantially higher odds of non-use compared with those not working (aOR 1.29, 95% CI 1.15–1.45; and aOR 1.31, 95% CI 1.07–1.59, respectively). A clear income gradient persisted, with the highest income quartile showing markedly higher odds of inpatient non-use (aOR 1.56, 95% CI 1.38–1.77). Rural residents were significantly more likely to not use JKN for inpatient services than urban residents (aOR 1.25, 95% CI 1.13–1.39). At the district level, greater hospital density was strongly protective against non-use (aOR 0.21, 95% CI 0.08–0.50), while primary care and doctor density were not independently associated; higher health worker density showed a modest protective effect (aOR 0.98, 95% CI 0.98–0.99). The intraclass correlation coefficient indicated that approximately 14% of the variance in inpatient non-use was attributable to between-district differences, underscoring the continued importance of contextual health system factors beyond individual characteristics.

### Sensitivity analysis

To avoid misclassification of insurance status, respondents reporting inactive JKN cards were excluded, as non-use in this group may reflect loss of eligibility rather than barriers to utilisation; corresponding results are presented in Table S1 and Table S2 in the Supplementary Files. Restricting the analysis to respondents with active JKN membership produced patterns that were largely consistent with the full sample while clarifying that non-use primarily reflects behavioural and structural constraints rather than administrative ineligibility. For outpatient care, associations remained robust: sex was not related to non-use, strong age gradients persisted with substantially lower non-use among adults and older persons, and higher educational attainment remained protective. Employment status continued to show the strongest effects, with self-employed, casual, and unpaid family workers consistently more likely to not use JKN, while gradients by household size, income, and rural residence were unchanged. Districts with higher hospital and primary care facility density continued to exhibit lower outpatient non-use, indicating persistent supply-side constraints even among insured members. For inpatient care, excluding inactive cards attenuated most sociodemographic gradients, with no independent associations for education and more limited employment effects, while the income gradient strengthened markedly and rural residence remained a significant risk factor. Notably, respondents reporting health problems affecting daily activities were less likely to not use JKN for inpatient services, suggesting that clinical need outweighs other barriers once hospitalisation is required.

## Discussion

This study provides the first nationally representative description of health insurance underutilisation among insured individuals in Indonesia, using recent post-pandemic data. Despite near-universal enrolment in JKN, a substantial proportion of insured respondents did not activate their insurance when seeking care, approximately half for outpatient services and one in ten for inpatient services. This contrast is important in itself and reflects well-established differences in care-seeking pathways: outpatient care is more discretionary, routine, and sensitive to administrative burden, whereas inpatient care is typically driven by clinical urgency and higher financial stakes [4, 7]. Although nationally comparable estimates from earlier years are limited, these magnitudes are broadly consistent with prior studies showing higher insurance activation for hospitalisation than for ambulatory care, both before and after the introduction of JKN [13, 19]. For instance, Pratiwi and colleagues found that inpatient service use is 135% higher for insured individuals compared to the uninsured, after controlling for district health status and service access, while outpatient service use increases by 25% for insured individuals reporting symptoms [19]. Taken together, this evidence suggests that underutilisation, particularly for outpatient care, has remained a persistent feature of Indonesia’s UHC system rather than a transient post-pandemic anomaly.

### Characterising inequities in insurance use among the insured

A key contribution of this study is to distinguish inequities in the use of health insurance among insured individuals from more commonly studied inequalities in healthcare utilisation in the general population. Our findings demonstrate that underutilisation of JKN is patterned by socioeconomic position, labour-market status, and geography. Rural residents, individuals engaged in informal employment, and those in higher household expenditure groups were consistently more likely to bypass JKN despite being covered. This pattern has been observed in prior studies in China [20], Laos [21] and Thailand [22], showing that insurance coverage does not automatically translate into effective use, particularly where service availability, administrative complexity, and opportunity costs remain salient.

This finding suggests that underutilisation reflects a combination of structural constraints (such as uneven facility distribution and administrative burden), behavioural responses, and perceived trade-offs between time, income, and quality of care, rather than lack of insurance coverage per se. Similar mechanisms have been documented in settings where insured individuals selectively bypass public insurance schemes in favour of out-of-pocket or private care when transaction costs or perceived quality gaps are high [23, 24].

Educational attainment emerged as a protective factor for outpatient, but not inpatient, insurance use, underscoring the role of health and administrative literacy in navigating referral rules, documentation requirements, and provider choice for routine services. Prior studies in Indonesia have likewise shown that lower educational attainment is associated with reduced ability to navigate insurance processes and lower utilisation of outpatient and preventive care [10, 25]. Health literacy is a key determinant of an individual’s ability to understand and make use of healthcare systems, including health insurance. In Indonesia, individuals with lower educational attainment often struggle to understand the options and benefits of health insurance, which can result in reduced healthcare utilisation [26]. Baker defines health literacy as the skills required to obtain, process, and understand basic health information for making informed health decisions [27]. Low levels of education directly correlate with inadequate health literacy, which leads to difficulties in managing health-related tasks and comprehending complex insurance materials.

In contrast, inpatient utilisation appeared less socially stratified, except by income and rural residence, reinforcing the idea that clinical severity and acute need can override some behavioural and administrative barriers once hospitalisation becomes unavoidable. This distinction between outpatient and inpatient care aligns with broader evidence that discretionary services are more sensitive to socioeconomic and administrative barriers, whereas high-acuity care is more likely to be accessed regardless of insurance frictions [4, 7].

### Employment, informality, and effective access

In this study, employment status emerged as one of the strongest correlates of JKN underutilisation, particularly for outpatient care. Self-employed individuals, casual workers, and unpaid family workers—groups closely aligned with informal employment—were substantially more likely to forgo insurance use than those who were formally employed or economically inactive. A substantial barrier for informal workers is the lack of awareness and understanding of health insurance products and benefits. Research by Kusi and collegues emphasises that low levels of education and understanding of the insurance concept among informal sector workers often result in a reluctance to enrol in health insurance plans [28]. The complexity of health insurance policies can deter informal workers, who may not fully recognise the advantages these plans offer, leading to low enrollment and utilisation rates. Furthermore, Kimani et al. demonstrate that inadequate mechanisms for collecting contributions from informal-sector workers impede their participation in public health insurance programs, thereby exacerbating their vulnerability [28]. Similarly, Amu et al. found that, despite the potential of health insurance as a financial protection mechanism, many informal-sector workers in sub-Saharan Africa remain unaware of its benefits [29]. This unawareness deters them from seeking healthcare services, even when they are technically insured.

Although JKN participant type (subsidised PBI versus contributory non-PBI) cannot be directly identified in SUSENAS, employment status and household economic position are closely linked to PBI eligibility in practice and therefore provide an indirect lens on this distinction. These variables should be interpreted as proxies rather than direct measures of subsidy status, and the observed gradients in underutilisation likely reflect a combination of differences in subsidy design, labour-market precarity, and broader structural barriers to effective access. From a policy perspective, these findings highlight a critical gap in Indonesia’s UHC architecture: while enrolment mechanisms have successfully expanded coverage among informal workers, effective access remains constrained by irregular incomes, administrative complexity, opportunity costs, and weaker attachment to formal health system routines. Similar dynamics have been observed in other UHC settings, where informal employment limits insured individuals’ ability to navigate referral systems, comply with administrative requirements, or prioritise preventive and outpatient care despite nominal coverage [30]. Addressing underutilisation among informal workers, therefore, requires not only maintaining coverage but also simplifying procedures, strengthening frontline service responsiveness, and tailoring insurance literacy and engagement strategies to precarious labour contexts.

### Health system context and reported barriers

At the district level, we found that higher hospital density and greater availability of health workers were associated with lower odds of JKN non-use, particularly for inpatient care. This pattern suggests that supply-side constraints, relating to the availability, accessibility, and distribution of health services, continue to shape effective access even among insured populations. Similar findings have been documented in Indonesia and other LMICs, where insufficient health workforce density and uneven facility distribution limit the translation of insurance coverage into effective service use [23, 31, 32]. Evidence from comparative settings further indicates that expanding insurance coverage without investments in health workforce and service capacity can result in persistent underutilisation or shifting care-seeking patterns, particularly between ambulatory and inpatient services [30, 33]. These findings reinforce the importance of aligning universal health coverage reforms with active stewardship of human resources for health and facility readiness across geographic areas.

Importantly, the reported reasons for non-use, such as long waiting times, inactive insurance cards, and administrative difficulties, are self-reported and collected only among respondents who did not use JKN in this study. As such, they reflect perceived and experienced barriers at the point of care rather than objectively verified measures of system performance. Prior research on effective coverage similarly emphasises that user-reported barriers capture critical dimensions of service acceptability and administrative friction that are often invisible in routine system indicators [34, 35].

### Strengths and limitations

This study has several key strengths. It provides one of the first nationally representative analyses of health insurance underutilisation in Indonesia, using microdata from the 2023 National Socio-Economic Survey (SUSENAS). The large analytical samples for outpatient (84,072) and inpatient (32,650) care ensure strong statistical power and broad district-level representativeness. By extending Andersen’s Behavioral Model to explicitly incorporate prohibiting factors, the study offers a theory-informed assessment of how behavioural, socioeconomic, and systemic barriers shape insurance use. In addition, the use of random-effects logistic regression allows contextual health system influences at the district level to be accounted for alongside individual characteristics.

However, several limitations should be considered when interpreting the findings of this study. First, the analysis is descriptive and cross-sectional; it is not intended to establish causal relationships but to characterise patterns of JKN underutilisation, the populations most affected, and the contexts in which non-use occurs. Second, the reported “reasons for non-use” (e.g. long waiting times, inactive insurance cards, and administrative difficulties) are self-reported and observed only among respondents who did not use JKN. As such, they reflect perceived barriers at the point of care rather than objectively measured aspects of health system performance. Third, socioeconomic position was proxied by per capita household expenditure, a widely used measure in Indonesian socioeconomic and health research, but not without limitations. Because healthcare spending contributes to total household expenditure, observed differences between users and non-users may partly reflect healthcare-related costs rather than underlying long-term economic status. Although expenditure-based measures are designed to capture broader consumption patterns rather than short-term shocks, some residual endogeneity cannot be ruled out. Finally, although SUSENAS is collected annually, the present analysis focuses on 2023 data, providing a snapshot of insurance utilisation during the post–COVID-19 recovery period. Temporal analyses examining changes in underutilisation before and after the pandemic were beyond the scope of this study but represent an important direction for future research using pooled or longitudinal data.

### Policy implications

Taken together, the findings reveal “hidden inequities” within Indonesia’s UHC system: enrolment does not automatically translate into effective use. These inequities appear to be driven less by eligibility and more by labour-market precarity, uneven rural service availability, and administrative burden. Policy responses must therefore move beyond coverage expansion toward ensuring utilisation equity in practice. Priority actions include simplifying administrative procedures (such as card activation, referral, and verification processes), strengthening frontline service capacity and provider responsiveness, particularly in rural and underserved areas, and enhancing community-level health insurance literacy among informal workers. In addition, routine monitoring of health insurance underutilisation should be incorporated into UHC performance frameworks to ensure that progress is assessed not only by enrolment but by effective access and use.

## Conclusions

This study demonstrates that achieving near-universal health insurance enrolment in Indonesia has not been sufficient to ensure equitable use of care. Persistent underutilisation of JKN among insured individuals, particularly for outpatient services, signals that important administrative, structural, and behavioural barriers remain embedded within the health system. These gaps disproportionately affect rural residents, informal-sector workers, and socioeconomically disadvantaged groups, revealing inequities that are obscured when UHC performance is assessed solely through coverage indicators.

Policy responses should therefore prioritise making insurance usable in practice. First, administrative simplification is essential, including faster card activation, streamlined referral and verification processes, and improved digital interoperability at the point of care. Second, strengthening frontline service capacity, especially in rural areas, through adequate staffing, facility availability, and reduced waiting times is critical to lowering the opportunity costs of using JKN. Third, targeted community-based insurance literacy and engagement strategies are needed to support insured individuals, particularly informal workers, in navigating entitlements and procedures.

Finally, routine monitoring of health insurance underutilisation should be integrated into national UHC evaluation frameworks as a core equity indicator. Shifting policy attention from enrolment alone to effective utilisation will be essential for ensuring that Indonesia’s universal health coverage reforms deliver real and equitable health gains.

## Supplementary Information

Supplementary Material 1.

## Data Availability

The data that support the findings of this study are available from the Indonesian Central Bureau of Statistics (Badan Pusat Statistik, BPS). The 2023 SUSENAS microdata can be accessed upon request through the BPS official data portal at https://www.bps.go.id. The data are not publicly available without registration, but are accessible to researchers upon reasonable request to BPS.

## Abbreviations

UHC: Universal Health Coverage
JKN: Jaminan Kesehatan Nasional (National Health Insurance)
PBI: Penerima Bantuan Iuran (contribution assistance recipients)
SUSENAS: Survei Sosial Ekonomi Nasional (National Socioeconomic Survey)
BPS: Badan Pusat Statistik (Indonesian Central Bureau of Statistics)
LMICs: Low- and Middle-Income Countries
aOR: Adjusted Odds Ratio
OR: Odds Ratio
ICC: Intraclass Correlation Coefficient
CI: Confidence Interval
EA: Enumeration Area
