# Supplementary material for: Hidden inequities in universal health coverage: determinants of health insurance underutilization in Indonesia
Source: BMC Public Health. 2026 Mar 11;26:1265. doi: 10.1186/s12889-026-26841-3 (PMC13088535; doi:10.1186/s12889-026-26841-3)
Supplement: Supplementary file 1 — Supplementary Material 1. [file 12889_2026_26841_MOESM1_ESM.docx]

**Figure S1.** Sample selection flow diagram showing inclusion, exclusions, and final analytical sample

**
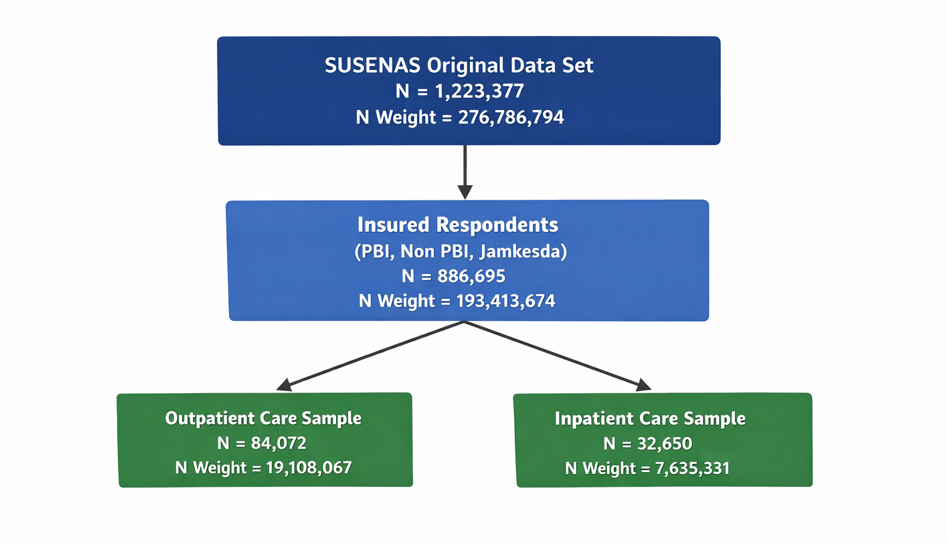
**

**Sensitivity Analysis**

To avoid misclassification of insurance status, respondents with inactive JKN cards were excluded, as non-use among this group may reflect loss of eligibility rather than barriers to utilisation. Individuals reporting inactive JKN cards were excluded from the analysis, as they may not be technically considered insured at the point of service use. Exclusion of inactive card holders ensures that the outcome reflects behavioural and access-related non-use of JKN benefits, rather than administrative loss of eligibility.

**Table S1**. Adjusted random-effects logistic regression of factors associated with non-use of JKN insurance among the outpatient sample after respondents with JKN card inactive removed (N = 83,097)

| Domain | Predictor | Category / Unit | aOR (95% CI) | p-value |
| --- | --- | --- | --- | --- |
| Individual characteristics | Sex | Female (vs male) | 0.99 (0.96–1.03) | 0.705 |
|  | Age group† | Infants (<1 year) | 1.00 (ref) | — |
|  |  | Children (1–9 years) | 0.99 (0.93–1.07) | 0.890 |
|  |  | Early adolescent (10–14 years) | 0.89 (0.82–0.96) | 0.003 |
|  |  | Late adolescent (15–19 years) | 1.02 (0.92–1.13) | 0.687 |
|  |  | Young adult (20–24 years) | 0.87 (0.78–0.98) | 0.019 |
|  |  | Adult (25–59 years) | 0.65 (0.60–0.69) | <0.001 |
|  |  | Older person (60+ years) | 0.58 (0.54–0.62) | <0.001 |
|  | Education† | Elementary or less | 1.00 (ref) | — |
|  |  | Junior secondary | 0.88 (0.84–0.93) | <0.001 |
|  |  | Senior secondary | 0.78 (0.74–0.82) | <0.001 |
|  |  | Diploma | 0.65 (0.56–0.75) | <0.001 |
|  |  | University | 0.77 (0.71–0.83) | <0.001 |
|  | Employment† | Not working | 1.00 (ref) | — |
|  |  | Self-employed | 1.42 (1.35–1.49) | <0.001 |
|  |  | Employee | 1.40 (1.32–1.48) | <0.001 |
|  |  | Casual worker | 1.52 (1.39–1.67) | <0.001 |
|  |  | Unpaid family worker | 1.41 (1.30–1.52) | <0.001 |
| Household characteristics | Household size | Per additional member | 1.03 (1.02–1.05) | <0.001 |
|  | Income per capita quartile† | Q1 (lowest) | 1.00 (ref) | — |
|  |  | Q2 | 1.17 (1.11–1.22) | <0.001 |
|  |  | Q3 | 1.15 (1.10–1.21) | <0.001 |
|  |  | Q4 (highest) | 1.37 (1.30–1.44) | <0.001 |
| Contextual factors | Residence | Rural (vs urban) | 1.48 (1.42–1.54) | <0.001 |
|  | Health need | Health problem affecting daily activities | 1.05 (1.01–1.09) | 0.006 |
| Health system supply (district level) | Hospital density | Per unit increase | 0.30 (0.13–0.72) | 0.007 |
|  | Primary care density | Per unit increase | 0.86 (0.82–0.90) | <0.001 |
|  | Doctor density | Per unit increase | 0.98 (0.94–1.03) | 0.539 |
|  | Health worker density | Per unit increase | 0.99 (0.99–1.00) | 0.015 |

Notes: Outcome: non-use of JKN insurance. † Reference categories: age group = infants; education = elementary or less; employment = not working; income = lowest quartile (Q1). Models estimated using random-effects logistic regression with individuals clustered within 514 districts. Model A: unadjusted random-effects logistic regression (one predictor at a time). Model B: fully adjusted random-effects logistic regression including all covariates shown. Model B statistics: Wald χ²=984.83; intraclass correlation (ρ)=0.205 (95% CI 0.182–0.229); likelihood-ratio test for random effects p<0.001. OR, odds ratio; aOR, adjusted odds ratio; CI, confidence interval.

Among insured respondents with active JKN membership, non-use of JKN for outpatient care remained strongly patterned by socioeconomic position, employment status, and local health system context. Sex was not associated with non-use after adjustment (female vs male: aOR 0.99, 95% CI 0.96–1.03). Clear age gradients were evident: compared with infants, adults aged 25–59 years and older persons were substantially less likely to not use JKN (aOR 0.65, 95% CI 0.60–0.69; and aOR 0.58, 95% CI 0.54–0.62), with similar protective effects observed among early adolescents and young adults. Higher educational attainment was consistently protective, including senior secondary (aOR 0.78, 95% CI 0.74–0.82) and university education (aOR 0.77, 95% CI 0.71–0.83). In contrast, employment status showed the strongest associations: self-employed individuals, employees, casual workers, and unpaid family workers all had markedly higher odds of non-use than those not working (aORs 1.40–1.52). Non-use increased with household size (aOR 1.03 per additional member, 95% CI 1.02–1.05) and across income quartiles, peaking in the highest quartile (aOR 1.37, 95% CI 1.30–1.44). Rural residence was strongly associated with non-use (aOR 1.48, 95% CI 1.42–1.54), and respondents reporting health problems affecting daily activities were slightly more likely to not use JKN (aOR 1.05, 95% CI 1.01–1.09). At the district level, higher hospital and primary care facility density were associated with lower non-use, whereas doctor density showed no independent association.

**Table S2.** Adjusted random-effects logistic regression of factors associated with non-use of JKN insurance among the inpatient sample after respondents with JKN card inactive removed (N = 32,342)

| Domain | Predictor | Category / Unit | aOR (95% CI) | p-value |
| --- | --- | --- | --- | --- |
| Individual characteristics | Sex | Female (vs male) | 0.98 (0.89–1.07) | 0.643 |
|  | Age group† | Infants (<1 year) | 1.00 (ref) | — |
|  |  | Children (1–9 years) | 1.02 (0.81–1.28) | 0.896 |
|  |  | Early adolescent (10–14 years) | 0.91 (0.69–1.19) | 0.490 |
|  |  | Late adolescent (15–19 years) | 1.20 (0.93–1.55) | 0.163 |
|  |  | Young adult (20–24 years) | 0.99 (0.77–1.26) | 0.923 |
|  |  | Adult (25–59 years) | 0.90 (0.75–1.09) | 0.297 |
|  |  | Older person (60+ years) | 0.82 (0.68–0.99) | 0.038 |
|  | Education† | Elementary or less (ref) | 1.00 | — |
|  |  | Junior secondary | 0.98 (0.86–1.12) | 0.800 |
|  |  | Senior secondary | 0.97 (0.86–1.10) | 0.672 |
|  |  | Diploma | 0.81 (0.60–1.10) | 0.185 |
|  |  | University | 0.93 (0.78–1.10) | 0.378 |
|  | Employment† | Not working (ref) | 1.00 | — |
|  |  | Self-employed | 1.28 (1.13–1.45) | <0.001 |
|  |  | Employee | 0.94 (0.82–1.09) | 0.438 |
|  |  | Casual worker | 1.23 (0.93–1.62) | 0.145 |
|  |  | Unpaid family worker | 1.37 (1.12–1.69) | 0.003 |
| Household characteristics | Household size | Per additional member | 1.02 (0.99–1.05) | 0.205 |
|  | Income per capita quartile† | Q1 (lowest) | 1.00 | — |
|  |  | Q2 | 1.12 (0.99–1.27) | 0.076 |
|  |  | Q3 | 1.17 (1.03–1.33) | 0.017 |
|  |  | Q4 (highest) | 1.65 (1.45–1.88) | <0.001 |
| Contextual factors | Residence | Rural (vs urban) | 1.27 (1.14–1.41) | <0.001 |
|  | Health need | Health problem affecting daily activities | 0.88 (0.80–0.96) | 0.003 |
| Health system supply (district level) | Hospital density | Per unit increase | 0.24 (0.10–0.61) | 0.003 |
|  | Primary care density | Per unit increase | 1.00 (0.95–1.05) | 0.952 |
|  | Doctor density | Per unit increase | 0.98 (0.93–1.03) | 0.358 |
|  | Health worker density | Per unit increase | 0.98 (0.97–0.99) | <0.001 |

Notes: Outcome: non-use of JKN insurance. † Reference categories: age group = infants; education = elementary or less; employment = not working; income = lowest quartile (Q1). Models estimated using random-effects logistic regression with individuals clustered within 506 districts. Model A: unadjusted random-effects logistic regression (one predictor at a time). Model B: fully adjusted random-effects logistic regression including all covariates shown. Model B statistics: Wald χ²= 228.73; intraclass correlation (ρ)=0.140 (95% CI 0.116–0.167); likelihood-ratio test for random effects p<0.001. OR, odds ratio; aOR, adjusted odds ratio; CI, confidence interval.

Among insured respondents with active JKN membership, patterns of non-use for inpatient care differed from those observed for outpatient services and were generally less strongly structured by individual sociodemographic characteristics. Sex was not associated with inpatient non-use after adjustment (female vs male: aOR 0.98, 95% CI 0.89–1.07). Age gradients were weaker, with most age groups showing no significant differences relative to infants; only older persons aged 60 years and above were modestly less likely to not use JKN for inpatient care (aOR 0.82, 95% CI 0.68–0.99). Educational attainment was not independently associated with inpatient non-use, with no clear protective gradient observed across education levels. Employment status showed selective effects: self-employed individuals and unpaid family workers had higher odds of inpatient non-use compared with those not working (aOR 1.28, 95% CI 1.13–1.45; and aOR 1.37, 95% CI 1.12–1.69), while employees and casual workers did not differ significantly. Household size was not associated with inpatient non-use, but a clear income gradient was evident, with the highest income quartile showing substantially higher odds (aOR 1.65, 95% CI 1.45–1.88). Rural residents were more likely to not use JKN for inpatient care (aOR 1.27, 95% CI 1.14–1.41). In contrast to outpatient care, respondents reporting health problems affecting daily activities were less likely to not use JKN (aOR 0.88, 95% CI 0.80–0.96). At the district level, higher hospital density was strongly associated with lower inpatient non-use (aOR 0.24, 95% CI 0.10–0.61), while primary care and doctor density were not associated; higher health worker density showed a small but significant protective effect.

**Table S3**. Average marginal effects (dy/dx) from multilevel logistic regression models of factors associated with non-use of JKN insurance among outpatient samples.

| Domain | Predictor | Category / Unit | AME (dy/dx) | 95% CI | p-value |
| --- | --- | --- | --- | --- | --- |
| Individual characteristics | Sex | Female (vs male) | −0.001 | −0.007 to 0.005 | 0.799 |
|  | Age group† | Infants (<1 year) | Ref | — | — |
|  |  | Children (1–9 years) | −0.000 | −0.014 to 0.014 | 0.987 |
|  |  | Early adolescent (10–14 years) | −0.023 | −0.038 to −0.008 | 0.003 |
|  |  | Late adolescent (15–19 years) | 0.005 | −0.015 to 0.024 | 0.640 |
|  |  | Young adult (20–24 years) | −0.025 | −0.046 to −0.003 | 0.024 |
|  |  | Adult (25–59 years) | −0.079 | −0.092 to −0.066 | <0.001 |
|  |  | Older person (60+ years) | −0.101 | −0.113 to −0.088 | <0.001 |
|  | Education† | Elementary or less | Ref | — | — |
|  |  | Junior secondary | −0.021 | −0.030 to −0.012 | <0.001 |
|  |  | Senior secondary | −0.044 | −0.053 to −0.035 | <0.001 |
|  |  | Diploma | −0.075 | −0.099 to −0.051 | <0.001 |
|  |  | University | −0.047 | −0.060 to −0.033 | <0.001 |
|  | Employment† | Not working | Ref | — | — |
|  |  | Self-employed | 0.064 | 0.055 to 0.073 | <0.001 |
|  |  | Employee | 0.059 | 0.048 to 0.069 | <0.001 |
|  |  | Casual worker | 0.078 | 0.061 to 0.096 | <0.001 |
|  |  | Unpaid family worker | 0.063 | 0.049 to 0.077 | <0.001 |
| Household characteristics | Household size | Per additional member | 0.006 | 0.004 to 0.008 | <0.001 |
|  | Income per capita quartile† | Q1 (lowest) | Ref | — | — |
|  |  | Q2 | 0.025 | 0.017 to 0.034 | <0.001 |
|  |  | Q3 | 0.023 | 0.015 to 0.031 | <0.001 |
|  |  | Q4 (highest) | 0.052 | 0.043 to 0.061 | <0.001 |
| Contextual factors | Residence | Rural (vs urban) | 0.069 | 0.062 to 0.077 | <0.001 |
|  | Health need | Health problem affecting daily activities | 0.009 | 0.003 to 0.015 | 0.003 |
| Health system supply (district) | Hospital density | Per unit increase | −0.228 | −0.381 to −0.075 | 0.003 |
|  | Primary care density | Per unit increase | −0.027 | −0.036 to −0.019 | <0.001 |
|  | Doctor density | Per unit increase | −0.003 | −0.011 to 0.006 | 0.525 |
|  | Health worker density | Per unit increase | −0.001 | −0.002 to −0.000 | 0.015 |

Notes: dy/dx = average marginal effect (absolute percentage-point change in probability of non-use of JKN). 95% CI shown as Lower and Upper bounds in separate columns. † Reference categories: infants (age), elementary education, not working (employment), income quartile 1 (lowest).

The marginal effects analysis shows that non-use of JKN for outpatient care among insured respondents is driven primarily by age, socioeconomic position, employment status, place of residence, and local health system capacity rather than sex. Sex was not associated with non-use, while clear age gradients were evident: compared with infants, adults aged 25–59 years and older persons had substantially lower probabilities of non-use, by 7.9 and 10.1 percentage points respectively, with smaller but significant reductions also observed among early adolescents and young adults. Higher educational attainment was consistently protective, with university education associated with a 4.7 percentage point lower probability of non-use compared with Elementary or less. In contrast, employment status showed the largest increases in non-use, particularly among casual workers, self-employed individuals, and unpaid family workers, whose probabilities of non-use were 6–8 percentage points higher than those not working. Non-use also increased steadily with household size and across income quartiles, reaching a 5.2 percentage point higher probability among the highest-income households. Rural residence was strongly associated with non-use, increasing the probability by 6.9 percentage points. At the district level, higher hospital and primary care density substantially reduced the probability of non-use, while doctor density showed no independent effect, indicating that facility availability rather than personnel numbers plays a more critical role in shaping outpatient utilisation among insured populations.

**Table S4**. Average marginal effects (dy/dx) from multilevel logistic regression models of factors associated with non-use of JKN insurance among inpatient samples.

| Domain | Predictor | Category / Unit | dy/dx | 95% CI | p-value |
| --- | --- | --- | --- | --- | --- |
| Individual characteristics | Sex | Female (vs male) | −0.002 | −0.009 to 0.005 | 0.501 |
|  | Age group† | Children (1–9 yrs) | 0.001 | −0.017 to 0.020 | 0.876 |
|  |  | Early adolescent (10–14 yrs) | −0.008 | −0.028 to 0.012 | 0.428 |
|  |  | Late adolescent (15–19 yrs) | 0.018 | −0.004 to 0.039 | 0.116 |
|  |  | Young adult (20–24 yrs) | −0.005 | −0.025 to 0.014 | 0.577 |
|  |  | Adult (25–59 yrs) | −0.009 | −0.024 to 0.006 | 0.225 |
|  |  | Older person (≥60 yrs) | −0.016 | −0.031 to −0.002 | 0.023 |
| Education† |  | Junior secondary | 0.001 | −0.009 to 0.011 | 0.793 |
|  |  | Senior secondary | −0.001 | −0.010 to 0.008 | 0.822 |
|  |  | Diploma | −0.017 | −0.037 to 0.003 | 0.099 |
|  |  | University | −0.007 | −0.019 to 0.006 | 0.282 |
| Employment† |  | Self-employed | 0.021 | 0.011 to 0.031 | <0.001 |
|  |  | Employee | −0.005 | −0.015 to 0.005 | 0.299 |
|  |  | Casual worker | 0.018 | −0.005 to 0.041 | 0.122 |
|  |  | Unpaid family worker | 0.022 | 0.004 to 0.040 | 0.016 |
| Household characteristics | Household size | Per additional member | 0.001 | −0.001 to 0.004 | 0.227 |
|  | Income quartile† | Q2 | 0.010 | 0.001 to 0.018 | 0.027 |
|  |  | Q3 | 0.009 | 0.000 to 0.017 | 0.047 |
|  |  | Q4 (highest) | 0.036 | 0.026 to 0.046 | <0.001 |
| Contextual factors | Residence | Rural (vs urban) | 0.018 | 0.010 to 0.025 | <0.001 |
|  | Health need | Health problem affecting daily activities | −0.010 | −0.016 to −0.003 | 0.003 |
| Health system supply (district) | Hospital density | Per unit increase | −0.123 | −0.192 to −0.053 | 0.001 |
|  | Primary care density | Per unit increase | −0.000 | −0.004 to 0.004 | 0.876 |
|  | Doctor density | Per unit increase | −0.002 | −0.006 to 0.002 | 0.248 |
|  | Health worker density | Per unit increase | −0.001 | −0.002 to −0.001 | <0.001 |

Notes: dy/dx = average marginal effect (absolute percentage-point change in probability of non-use of JKN). 95% CI shown as Lower and Upper bounds in separate columns. † Reference categories: infants (age), elementary or less, not working (employment), income quartile 1 (lowest).

The marginal effects analysis shows that non-use of JKN for inpatient care is shaped primarily by socioeconomic position, employment status, rural residence, and district-level health system capacity, rather than by most individual demographic characteristics. Sex and most age groups are not significantly associated with non-use, although older persons (≥60 years) are modestly less likely to bypass JKN, indicating greater reliance on insurance when hospitalisation is required. Educational attainment does not independently influence inpatient non-use, suggesting that schooling-related gradients observed in outpatient care do not extend to inpatient services. Employment status remains important: self-employed individuals and unpaid family workers have a higher probability of not using JKN, pointing to labour-market-related barriers or preferences even for higher-acuity care. A clear income gradient is evident, with progressively higher probabilities of non-use across income quartiles, particularly among the richest households, consistent with greater capacity to opt out of JKN. Rural residence is associated with higher inpatient non-use, highlighting persistent access and referral constraints outside urban areas. In contrast, respondents reporting health problems affecting daily activities are less likely to not use JKN, suggesting that clinical need overrides other barriers when hospital care is required. At the district level, greater hospital availability is strongly protective against non-use, and higher health worker density has a smaller but significant protective effect, underscoring the continued importance of local health system capacity in shaping insurance utilisation for inpatient services.

**Table S5. Random-effects logistic regression of factors associated with non-use of JKN insurance for outpatient care, including interaction between income and residence**

| Domain | Predictor | OR (95% CI) | p-value |
| --- | --- | --- | --- |
| Individual characteristics |  |  |  |
| Sex | Female (vs male) | 1.00 (0.96–1.03) | 0.806 |
| Age group† | Children (1–9 years) | 1.00 (0.93–1.07) | 0.992 |
|  | Early adolescent (10–14 years) | 0.88 (0.82–0.96) | 0.003 |
|  | Late adolescent (15–19 years) | 1.02 (0.93–1.13) | 0.654 |
|  | Young adult (20–24 years) | 0.88 (0.78–0.98) | 0.024 |
|  | Adult (25–59 years) | 0.65 (0.61–0.70) | <0.001 |
|  | Older person (60+ years) | 0.57 (0.54–0.61) | <0.001 |
| Education† | Junior secondary | 0.89 (0.85–0.94) | <0.001 |
|  | Senior secondary | 0.78 (0.74–0.82) | <0.001 |
|  | Diploma | 0.65 (0.56–0.76) | <0.001 |
|  | University | 0.77 (0.72–0.83) | <0.001 |
| Employment† | Self-employed | 1.43 (1.36–1.50) | <0.001 |
|  | Employee | 1.38 (1.30–1.47) | <0.001 |
|  | Casual worker | 1.54 (1.40–1.69) | <0.001 |
|  | Unpaid family worker | 1.42 (1.31–1.53) | <0.001 |
| Household characteristics |  |  |  |
| Household size | Per additional member | 1.03 (1.02–1.05) | <0.001 |
| Income quartile† | Q2 (vs Q1) | 1.13 (1.05–1.21) | 0.001 |
|  | Q3 | 1.11 (1.03–1.19) | 0.007 |
|  | Q4 (highest) | 1.27 (1.19–1.37) | <0.001 |
| Contextual factors |  |  |  |
| Residence | Rural (vs urban) | 1.41 (1.31–1.51) | <0.001 |
| Health need | Health complaint affecting daily activities | 1.05 (1.02–1.09) | 0.003 |
| Health system supply (district level) |  |  |  |
| Hospital density | Per unit increase | 0.28 (0.12–0.66) | 0.004 |
| Primary care density | Per unit increase | 0.86 (0.82–0.90) | <0.001 |
| Doctor density | Per unit increase | 0.98 (0.94–1.03) | 0.534 |
| Health worker density | Per unit increase | 0.99 (0.99–1.00) | 0.015 |
| Interaction effects |  |  |  |
| Income × residence | Q2 × rural | 1.04 (0.95–1.14) | 0.386 |
|  | Q3 × rural | 1.05 (0.95–1.15) | 0.327 |
|  | Q4 × rural | 1.09 (0.99–1.20) | 0.067 |

Notes: Districts (random intercepts): 514. σᵤ = 0.92 (95% CI 0.86–0.99). Intraclass correlation coefficient (ICC, ρ) = 0.21 (95% CI 0.18–0.23). Likelihood-ratio test of random effects: p < 0.001. Odds ratios (ORs) are reported with 95% confidence intervals. † Reference categories: infants (<1 year) for age, elementary or less, not working, income quartile 1, and urban residence.

After including interaction between income and residence indicates that the association between household income and non-use of JKN insurance is broadly consistent across urban and rural settings, with limited evidence of effect modification by place of residence. Although higher income was independently associated with greater odds of non-use, the income–residence interaction terms were generally small and statistically non-significant. Compared with the poorest urban group, rural residents in the second- and third-income quartiles did not exhibit materially different odds of non-use (Q2×rural: OR 1.04, 95% CI 0.95–1.14; Q3×rural: OR 1.05, 95% CI 0.95–1.15). The interaction for the highest income quartile suggested a modest amplification of non-use in rural areas (Q4×rural: OR 1.09, 95% CI 0.99–1.20), but this estimate narrowly crossed the conventional threshold for statistical significance. Taken together, these findings suggest that while both higher income and rural residence are independently associated with greater non-use of JKN, their effects are largely additive rather than multiplicative, with only weak evidence that high-income households in rural areas face distinct or compounded barriers beyond those observed for income or residence alone.

**Table S6. Random-effects logistic regression of factors associated with non-use of JKN insurance for inpatient care, including interaction between income and residence**

| Domain | Predictor | OR (95% CI) | p-value |
| --- | --- | --- | --- |
| Individual characteristics |  |  |  |
| Sex | Female (vs male) | 0.97 (0.89–1.06) | 0.517 |
| Age group† | Children (1–9 years) | 1.02 (0.82–1.27) | 0.861 |
|  | Early adolescents (10–14 years) | 0.90 (0.70–1.17) | 0.433 |
|  | Late adolescents (15–19 years) | 1.21 (0.95–1.54) | 0.118 |
|  | Young adults (20–24 years) | 0.94 (0.74–1.18) | 0.581 |
|  | Adults (25–59 years) | 0.89 (0.75–1.07) | 0.211 |
|  | Older persons (≥60 years) | 0.81 (0.68–0.96) | 0.018 |
| Education† | Junior secondary | 1.02 (0.90–1.15) | 0.803 |
|  | Senior secondary | 0.99 (0.88–1.12) | 0.862 |
|  | Diploma | 0.80 (0.59–1.07) | 0.133 |
|  | University | 0.92 (0.78–1.08) | 0.316 |
| Employment† | Self-employed | 1.29 (1.15–1.45) | <0.001 |
|  | Employee | 0.93 (0.81–1.07) | 0.298 |
|  | Casual worker | 1.25 (0.96–1.63) | 0.097 |
|  | Unpaid family worker | 1.31 (1.07–1.60) | 0.008 |
| Household characteristics |  |  |  |
| Household size | Per additional member | 1.02 (0.99–1.05) | 0.214 |
| Income quartile† | Q2 | 1.05 (0.87–1.26) | 0.614 |
|  | Q3 | 0.93 (0.77–1.12) | 0.456 |
|  | Q4 (highest) | 1.43 (1.20–1.71) | <0.001 |
| Contextual factors |  |  |  |
| Residence | Rural (vs urban) | 1.08 (0.90–1.30) | 0.395 |
| Health need | Health complaint affecting daily activities | 0.88 (0.81–0.96) | 0.002 |
| Health system supply (district level) |  |  |  |
| Hospital density | Per unit increase | 0.21 (0.09–0.51) | 0.001 |
| Primary care density | Per unit increase | 1.00 (0.95–1.05) | 0.887 |
| Doctor density | Per unit increase | 0.97 (0.93–1.02) | 0.258 |
| Health worker density | Per unit increase | 0.98 (0.98–0.99) | <0.001 |
| Interaction effects |  |  |  |
| Income × residence | Q2 × rural | 1.15 (0.90–1.46) | 0.265 |
|  | Q3 × rural | 1.39 (1.09–1.77) | 0.007 |
|  | Q4 × rural | 1.13 (0.89–1.44) | 0.301 |

Notes: Districts (random intercepts): 506. σᵤ = 0.73 (95% CI 0.66–0.81). Intraclass correlation coefficient (ICC, ρ) = 0.14 (95% CI 0.12–0.17). Likelihood-ratio test of random effects: p < 0.001. Odds ratios (ORs) are reported with 95% confidence intervals. † Reference categories: infants (<1 year) for age, elementary or less, not working, income quartile 1 (lowest), and urban residence.

After including interaction between income and residence indicates that the relationship between socioeconomic status and inpatient non-use of JKN varies by rural–urban context, although the effect is selective rather than uniform. In the adjusted model, most income–residence interaction terms were not statistically significant, indicating that rural residence does not systematically amplify income-related differences in inpatient non-use. However, a notable exception was observed among individuals in the third income quartile living in rural areas, who had significantly higher odds of not using JKN compared with their urban counterparts in the lowest income quartile (Q3 × rural: OR 1.39, 95% CI 1.09–1.77). This finding suggests a subgroup for whom moderate economic resources combined with rural residence may facilitate bypassing JKN for inpatient care, potentially through greater use of out-of-pocket payment or alternative arrangements despite geographic constraints. By contrast, the interaction for the highest income quartile in rural areas was not statistically significant, implying that the effect of high income on inpatient non-use operates largely independently of residence. Overall, the interaction analysis indicates limited but meaningful effect modification, highlighting that income-related opting out of JKN for inpatient services may be context-specific and particularly pronounced among middle-income populations in rural settings rather than among the poorest or the richest groups.

**Table S7.** Random-effects logistic regression of factors associated with non-use of JKN insurance among outpatient hospital visits (N = 15,684)

| Domain | Predictor | OR (95% CI) | p-value |
| --- | --- | --- | --- |
| Individual characteristics |  |  |  |
| Sex | Female (vs male) | 0.88 (0.77–1.00) | 0.046 |
| Age group† | Children (1–9 years) | 1.16 (0.84–1.60) | 0.372 |
|  | Early adolescent (10–14 years) | 0.77 (0.52–1.13) | 0.186 |
|  | Late adolescent (15–19 years) | 1.51 (1.04–2.20) | 0.032 |
|  | Young adult (20–24 years) | 0.66 (0.41–1.04) | 0.071 |
|  | Adult (25–59 years) | 0.46 (0.35–0.62) | <0.001 |
|  | Older person (60+ years) | 0.41 (0.31–0.54) | <0.001 |
| Education† | Junior secondary | 0.87 (0.72–1.07) | 0.184 |
|  | Senior secondary | 0.88 (0.73–1.06) | 0.177 |
|  | Diploma | 0.88 (0.58–1.34) | 0.552 |
|  | University | 1.03 (0.81–1.31) | 0.812 |
| Employment† | Self-employed | 1.63 (1.37–1.94) | <0.001 |
|  | Employee | 1.38 (1.11–1.70) | 0.003 |
|  | Casual worker | 1.50 (0.95–2.37) | 0.079 |
|  | Unpaid family worker | 1.68 (1.23–2.29) | 0.001 |
| Household characteristics |  |  |  |
| Household size | Per additional member | 1.01 (0.96–1.05) | 0.721 |
| Income quartile† | Q2 (vs Q1) | 1.23 (0.99–1.54) | 0.066 |
|  | Q3 | 1.26 (1.02–1.57) | 0.035 |
|  | Q4 (highest) | 1.72 (1.40–2.12) | <0.001 |
| Contextual factors |  |  |  |
| Residence | Rural (vs urban) | 1.61 (1.37–1.89) | <0.001 |
| Health need | Health complaint affecting daily activities | 1.24 (1.07–1.42) | 0.003 |
| Health system supply (district level) |  |  |  |
| Hospital density | Per unit increase | 0.55 (0.20–1.49) | 0.240 |
| Primary care density | Per unit increase | 0.99 (0.93–1.05) | 0.779 |
| Doctor density | Per unit increase | 0.97 (0.91–1.02) | 0.228 |
| Health worker density | Per unit increase | 0.98 (0.98–0.99) | <0.001 |

Notes: Random intercepts at district level (507 districts). σᵤ = 0.72 (95% CI 0.62–0.83). Intraclass correlation coefficient (ICC, ρ) = 0.14 (95% CI 0.10–0.17). Likelihood-ratio test of random effects: p < 0.001. Odds ratios (ORs) reported with 95% confidence intervals. † Reference categories: infants (<1 year) for age, elementary or less, not working, income quartile 1, and urban residence.

**Table S8.** Random-effects logistic regression of factors associated with non-use of JKN insurance among outpatient *Puskesmas* visits (N = 36,868)

| Domain | Predictor | OR (95% CI) | p-value |
| --- | --- | --- | --- |
| Individual characteristics |  |  |  |
| Sex | Female (vs male) | 0.99 (0.91–1.08) | 0.876 |
| Age group† | Children (1–9 years) | 0.85 (0.71–1.02) | 0.082 |
|  | Early adolescent (10–14 years) | 0.79 (0.65–0.97) | 0.024 |
|  | Late adolescent (15–19 years) | 0.95 (0.74–1.22) | 0.695 |
|  | Young adult (20–24 years) | 0.91 (0.68–1.20) | 0.494 |
|  | Adult (25–59 years) | 0.77 (0.64–0.92) | 0.004 |
|  | Older person (60+ years) | 0.67 (0.56–0.79) | <0.001 |
| Education† | Junior secondary | 0.90 (0.79–1.02) | 0.110 |
|  | Senior secondary | 0.82 (0.72–0.94) | 0.005 |
|  | Diploma | 0.77 (0.48–1.23) | 0.271 |
|  | University | 0.79 (0.63–0.99) | 0.043 |
| Employment† | Self-employed | 1.05 (0.93–1.19) | 0.445 |
|  | Employee | 1.10 (0.94–1.30) | 0.240 |
|  | Casual worker | 1.21 (0.97–1.52) | 0.094 |
|  | Unpaid family worker | 1.11 (0.93–1.33) | 0.242 |
| Household characteristics |  |  |  |
| Household size | Per additional member | 1.00 (0.97–1.04) | 0.752 |
| Income quartile† | Q2 (vs Q1) | 1.10 (0.98–1.23) | 0.095 |
|  | Q3 | 1.10 (0.98–1.23) | 0.122 |
|  | Q4 (highest) | 1.47 (1.29–1.68) | <0.001 |
| Contextual factors |  |  |  |
| Residence | Rural (vs urban) | 2.19 (1.96–2.46) | <0.001 |
| Health need | Health complaint affecting daily activities | 1.04 (0.96–1.14) | 0.343 |
| Health system supply (district level) |  |  |  |
| Hospital density | Per unit increase | 0.23 (0.07–0.73) | 0.013 |
| Primary care density | Per unit increase | 0.99 (0.93–1.05) | 0.772 |
| Doctor density | Per unit increase | 0.95 (0.88–1.02) | 0.153 |
| Health worker density | Per unit increase | 0.99 (0.98–0.99) | 0.001 |

Notes: Random intercepts at district level (514 districts). σᵤ = 1.10 (95% CI 1.01–1.19). Intraclass correlation coefficient (ICC, ρ) = 0.27 (95% CI 0.24–0.30). Likelihood-ratio test of random effects: p < 0.001. Odds ratios (ORs) are reported with 95% confidence intervals. † Reference categories: infants (<1 year) for age, elementary or less, not working, income quartile 1, and urban residence.

**Table S9.** Random-effects logistic regression of factors associated with non-use of JKN insurance among outpatient private clinics visits (N = 34,240)

| Domain | Predictor | OR (95% CI) | p-value |
| --- | --- | --- | --- |
| Individual characteristics |  |  |  |
| Sex | Female (vs male) | 0.98 (0.93–1.03) | 0.398 |
| Age group† | Children (1–9 years) | 1.02 (0.92–1.13) | 0.759 |
|  | Early adolescent (10–14 years) | 1.03 (0.91–1.16) | 0.649 |
|  | Late adolescent (15–19 years) | 1.41 (1.21–1.65) | <0.001 |
|  | Young adult (20–24 years) | 1.47 (1.23–1.76) | <0.001 |
|  | Adult (25–59 years) | 0.98 (0.88–1.09) | 0.682 |
|  | Older person (60+ years) | 0.84 (0.76–0.93) | 0.001 |
| Education† | Junior secondary | 0.88 (0.81–0.96) | 0.002 |
|  | Senior secondary | 0.68 (0.62–0.73) | <0.001 |
|  | Diploma | 0.50 (0.40–0.61) | <0.001 |
|  | University | 0.61 (0.54–0.68) | <0.001 |
| Employment† | Self-employed | 1.45 (1.34–1.57) | <0.001 |
|  | Employee | 1.11 (1.01–1.21) | 0.025 |
|  | Casual worker | 1.66 (1.41–1.95) | <0.001 |
|  | Unpaid family worker | 1.37 (1.20–1.56) | <0.001 |
| Household characteristics |  |  |  |
| Household size | Per additional member | 1.03 (1.01–1.05) | 0.005 |
| Income quartile† | Q2 (vs Q1) | 0.92 (0.85–0.99) | 0.031 |
|  | Q3 | 0.69 (0.64–0.74) | <0.001 |
|  | Q4 (highest) | 0.65 (0.60–0.70) | <0.001 |
| Contextual factors |  |  |  |
| Residence | Rural (vs urban) | 1.86 (1.75–1.98) | <0.001 |
| Health need | Health complaint affecting daily activities | 1.09 (1.04–1.15) | 0.001 |
| Health system supply (district level) |  |  |  |
| Hospital density | Per unit increase | 0.49 (0.23–1.06) | 0.072 |
| Primary care density | Per unit increase | 1.00 (0.95–1.05) | 0.980 |
| Doctor density | Per unit increase | 1.00 (0.96–1.04) | 0.929 |
| Health worker density | Per unit increase | 0.99 (0.99–1.00) | 0.004 |

Notes: Random intercepts at district level (498 districts). σᵤ = 0.74 (95% CI 0.68–0.80). Intraclass correlation coefficient (ICC, ρ) = 0.14 (95% CI 0.12–0.16). Likelihood-ratio test of random effects: p < 0.001. Odds ratios (ORs) are reported with 95% confidence intervals. † Reference categories: infants (<1 year) for age, elementary or less, not working, income quartile 1, and urban residence.

Across the three care settings—outpatient hospital only, Puskesmas only, and outpatient clinic only—the results show a consistent but context-specific pattern in the determinants of non-use of JKN insurance. Age gradients are evident in all settings, with late adolescents generally exhibiting higher odds of non-use, while older adults show substantially lower odds, particularly in hospital and clinic settings, indicating greater reliance on JKN among older populations when seeking care. Education displays a strong and monotonic protective effect, most pronounced in Puskesmas and clinic visits, where higher educational attainment is associated with markedly lower odds of non-use, suggesting that health literacy and administrative navigation play a larger role in primary and private outpatient care than in hospitals. Employment status is a robust risk factor across all three settings, especially for self-employed, casual, and unpaid family workers, who consistently exhibit higher odds of non-use compared with those not working—highlighting structural vulnerabilities among informal workers irrespective of provider type. Economic gradients differ by setting: higher income is associated with increased non-use in hospital-only outpatient care, while the opposite pattern is observed in clinics, where higher-income groups are significantly more likely to bypass JKN, consistent with greater willingness to pay out-of-pocket for speed or perceived quality. Rural residence strongly increases the odds of non-use in all models, with the largest effects in Puskesmas and clinic settings, underscoring persistent geographic barriers in insurance utilisation beyond service availability alone. Finally, health system supply factors show limited and inconsistent effects, with hospital and health-worker density occasionally reducing non-use but with smaller magnitudes than individual and household characteristics, indicating that demand-side and administrative factors dominate decisions to use JKN across outpatient settings, while the relative importance of socioeconomic gradients varies by type of provider.

**Table S10.** Random-effects logistic regression of factors associated with non-use of JKN insurance among inpatient hospital visits (N = 5,960)

| Domain | Predictor | OR (95% CI) | p-value |
| --- | --- | --- | --- |
| Individual characteristics |  |  |  |
| Sex | Female (vs male) | 1.02 (0.79–1.33) | 0.871 |
| Age group† | Children (1–9 years) | 1.53 (0.73–3.22) | 0.261 |
|  | Early adolescent (10–14 years) | 1.10 (0.46–2.60) | 0.834 |
|  | Late adolescent (15–19 years) | 1.39 (0.62–3.12) | 0.418 |
|  | Young adult (20–24 years) | 0.91 (0.37–2.26) | 0.838 |
|  | Adult (25–59 years) | 0.74 (0.40–1.37) | 0.341 |
|  | Older person (60+ years) | 0.81 (0.46–1.45) | 0.486 |
| Education† | Junior secondary | 1.21 (0.83–1.75) | 0.319 |
|  | Senior secondary | 1.21 (0.84–1.72) | 0.303 |
|  | Diploma | 0.68 (0.24–1.92) | 0.462 |
|  | University | 1.22 (0.75–1.98) | 0.420 |
| Employment† | Self-employed | 1.41 (1.01–1.97) | 0.046 |
|  | Employee | 0.80 (0.50–1.26) | 0.329 |
|  | Casual worker | 1.29 (0.53–3.11) | 0.575 |
|  | Unpaid family worker | 1.65 (0.87–3.15) | 0.127 |
| Household characteristics |  |  |  |
| Household size | Per additional member | 1.06 (0.98–1.15) | 0.159 |
| Income quartile† | Q2 (vs Q1) | 1.35 (0.87–2.08) | 0.180 |
|  | Q3 | 1.45 (0.95–2.21) | 0.087 |
|  | Q4 (highest) | 2.20 (1.46–3.31) | <0.001 |
| Contextual factors |  |  |  |
| Residence | Rural (vs urban) | 1.71 (1.27–2.31) | <0.001 |
| Health system supply (district level) |  |  |  |
| Hospital density | Per unit increase | 0.39 (0.07–2.08) | 0.273 |
| Primary care density | Per unit increase | 0.93 (0.84–1.04) | 0.232 |
| Doctor density | Per unit increase | 0.94 (0.86–1.04) | 0.243 |
| Health worker density | Per unit increase | 0.99 (0.98–1.00) | 0.082 |

Notes: Random intercepts at district level (492 districts). σᵤ = 0.67 (95% CI 0.48–0.94). Intraclass correlation coefficient (ICC, ρ) = 0.12 (95% CI 0.07–0.21). Likelihood-ratio test of random effects: p < 0.001. Odds ratios (ORs) are reported with 95% confidence intervals. † Reference categories: infants (<1 year) for age, elementary or less, not working, income quartile 1, and urban residence. Category health need omitted due to collinearity.

**Table S11.** Random-effects logistic regression of factors associated with non-use of JKN insurance among inpatient Puskesmas visits (N = 3,271)

| Domain | Predictor | OR (95% CI) | p-value |
| --- | --- | --- | --- |
| Individual characteristics |  |  |  |
| Sex | Female (vs male) | 0.86 (0.61–1.22) | 0.400 |
| Age group† | Children (1–9 years) | 1.10 (0.48–2.50) | 0.822 |
|  | Early adolescent (10–14 years) | 0.57 (0.18–1.83) | 0.343 |
|  | Late adolescent (15–19 years) | 1.44 (0.56–3.71) | 0.446 |
|  | Young adult (20–24 years) | 0.76 (0.25–2.35) | 0.635 |
|  | Adult (25–59 years) | 0.95 (0.49–1.84) | 0.878 |
|  | Older person (60+ years) | 0.82 (0.43–1.58) | 0.560 |
| Education† | Junior secondary | 1.14 (0.73–1.78) | 0.561 |
|  | Senior secondary | 0.77 (0.47–1.26) | 0.293 |
|  | Diploma | 0.69 (0.15–3.24) | 0.636 |
|  | University | 0.48 (0.19–1.23) | 0.127 |
| Employment† | Self-employed | 1.45 (0.95–2.20) | 0.083 |
|  | Employee | 1.60 (0.89–2.89) | 0.115 |
|  | Casual worker | 0.91 (0.30–2.74) | 0.869 |
|  | Unpaid family worker | 1.07 (0.52–2.21) | 0.847 |
| Household characteristics |  |  |  |
| Household size | Per additional member | 1.01 (0.90–1.13) | 0.836 |
| Income quartile† | Q2 (vs Q1) | 1.05 (0.70–1.58) | 0.804 |
|  | Q3 | 0.91 (0.60–1.40) | 0.681 |
|  | Q4 (highest) | 1.26 (0.78–2.05) | 0.341 |
| Contextual factors |  |  |  |
| Residence | Rural (vs urban) | 1.18 (0.80–1.72) | 0.406 |
| Health system supply (district level) |  |  |  |
| Hospital density | Per unit increase | 0.15 (0.02–1.36) | 0.092 |
| Primary care density | Per unit increase | 1.05 (0.94–1.17) | 0.392 |
| Doctor density | Per unit increase | 0.95 (0.82–1.09) | 0.458 |
| Health worker density | Per unit increase | 0.99 (0.97–1.00) | 0.069 |

Notes: Random intercepts at district level (482 districts). σᵤ = 0.83 (95% CI 0.59–1.18). Intraclass correlation coefficient (ICC, ρ) = 0.17 (95% CI 0.10–0.30). Likelihood-ratio test of random effects: p < 0.001. Odds ratios (ORs) are reported with 95% confidence intervals. † Reference categories: infants (<1 year) for age, elementary or less, not working, income quartile 1, and urban residence. Category health need omitted due to collinearity.

**Table S12.** Random-effects logistic regression of factors associated with non-use of JKN insurance among inpatient clinics visits (N = 2,575)

| Domain | Predictor | OR (95% CI) | p-value |
| --- | --- | --- | --- |
| Individual characteristics |  |  |  |
| Sex | Female (vs male) | 0.93 (0.72–1.19) | 0.552 |
| Age group† | Children (1–9 years) | 1.09 (0.61–1.93) | 0.773 |
|  | Early adolescent (10–14 years) | 0.96 (0.45–2.06) | 0.924 |
|  | Late adolescent (15–19 years) | 1.99 (0.97–4.09) | 0.061 |
|  | Young adult (20–24 years) | 1.11 (0.51–2.43) | 0.787 |
|  | Adult (25–59 years) | 1.18 (0.73–1.91) | 0.494 |
|  | Older person (60+ years) | 1.07 (0.67–1.68) | 0.784 |
| Education† | Junior secondary | 0.84 (0.58–1.22) | 0.351 |
|  | Senior secondary | 0.86 (0.61–1.23) | 0.412 |
|  | Diploma | 0.15 (0.02–1.16) | 0.068 |
|  | University | 0.75 (0.45–1.27) | 0.291 |
| Employment† | Self-employed | 1.17 (0.84–1.62) | 0.356 |
|  | Employee | 0.96 (0.63–1.48) | 0.868 |
|  | Casual worker | 1.35 (0.63–2.86) | 0.441 |
|  | Unpaid family worker | 1.52 (0.87–2.66) | 0.141 |
| Household characteristics |  |  |  |
| Household size | Per additional member | 0.97 (0.88–1.06) | 0.458 |
| Income quartile† | Q2 (vs Q1) | 0.76 (0.54–1.07) | 0.118 |
|  | Q3 | 0.82 (0.59–1.16) | 0.267 |
|  | Q4 (highest) | 1.00 (0.71–1.41) | 0.983 |
| Contextual factors |  |  |  |
| Residence | Rural (vs urban) | 1.17 (0.89–1.54) | 0.247 |
| Health system supply (district level) |  |  |  |
| Hospital density | Per unit increase | 0.05 (0.01–0.41) | 0.005 |
| Primary care density | Per unit increase | 1.00 (0.86–1.16) | 0.997 |
| Doctor density | Per unit increase | 1.01 (0.92–1.12) | 0.793 |
| Health worker density | Per unit increase | 0.97 (0.96–0.99) | <0.001 |

**Notes:** Random intercepts at district level (424 districts). σᵤ = 0.79 (95% CI 0.61–1.03). Intraclass correlation coefficient (ICC, ρ) = 0.16 (95% CI 0.10–0.24). Likelihood-ratio test of random effects: p < 0.001. Odds ratios (ORs) are reported with 95% confidence intervals. † Reference categories: infants (<1 year) for age, elementary or less, not working, income quartile 1, and urban residence. Category health need omitted due to collinearity.

Across the three inpatient settings—hospital, Puskesmas, and clinic—the results show a consistent but context-specific pattern in the determinants of non-use of JKN insurance. Rural residence is the most robust predictor in hospital inpatient care, where rural patients have substantially higher odds of not using JKN, suggesting persistent geographic and referral-related barriers for more complex care. In contrast, rural–urban differences are weaker and statistically non-significant in inpatient Puskesmas and clinic settings, indicating that primary-level inpatient services may be more geographically accessible. Household economic gradients are most evident in hospital inpatient visits, where individuals in the highest income quartile are significantly more likely to forgo JKN, consistent with selective opting-out or preference for non-JKN pathways when higher-cost services are involved; this gradient is attenuated and largely absent in Puskesmas and clinic inpatient care. Employment status shows elevated odds of non-use among informal and unpaid family workers in hospital settings, while these associations are weaker and imprecisely estimated in smaller Puskesmas and clinic samples. Across all three models, health system supply factors—particularly higher hospital density (where estimable) and greater health-worker density—are associated with lower odds of non-use, underscoring the importance of local service availability in enabling effective insurance utilisation. Finally, district-level random effects remain non-trivial across settings, indicating that unobserved contextual factors—such as local administrative practices or provider behaviour—continue to shape JKN use beyond individual socioeconomic characteristics.
